# Divergent fates of antigen-specific CD8^+^ T cell clones in mice with acute leukemia

**DOI:** 10.1016/j.celrep.2021.109991

**Published:** 2021-11-09

**Authors:** Xiufen Chen, Brendan W. MacNabb, Blake Flood, Bruce R. Blazar, Justin Kline

**Affiliations:** 1 Department of Medicine, University of Chicago, Chicago, IL, USA; 2 Committee on Immunology, University of Chicago, Chicago, IL, USA; 3 Department of Pediatrics, Division of Blood and Marrow Transplantation, University of Minnesota, Minneapolis, MN, USA; 4 University of Chicago Comprehensive Cancer Center, Chicago, IL, USA; 5 Lead contact

## Abstract

The existence of a dysfunctional CD8^+^ T cell state in cancer is well established. However, the degree to which CD8^+^ T cell fates are influenced by the context in which they encounter cognate tumor antigen is less clear. We previously demonstrated that CD8^+^ T cells reactive to a model leukemia antigen were deleted by antigen cross-presenting type 1 conventional dendritic cells (cDC1s). Here, through a study of T cell receptor (TCR) transgenic CD8^+^ T cells (TCR_Tg101_) reactive to a native C1498 leukemia cell antigen, we uncover a different mode of T cell tolerance in which TCR_Tg101_ undergo progressive expansion and differentiation into an exhausted state. Antigen encounter by TCR_Tg101_ requires leukemia cell major histocompatibility complex (MHC)-I expression and is independent of DCs, implying that leukemia cells directly mediate the exhausted TCR_Tg101_ phenotype. Collectively, our data reveal that leukemia antigens are presented to CD8^+^ T cells via discrete pathways, leading to distinct tolerant states.

## In brief

In order to track antigen-specific CD8^+^ T cell fates in leukemia-bearing hosts, Chen et al. generate a leukemia-specific TCR transgenic mouse (Tg101). They find that leukemia-specific CD8^+^ T cells expand and acquire a profoundly dysfunctional phenotype, which is mediated through direct antigen presentation by leukemia cells.

## Graphical Abstract

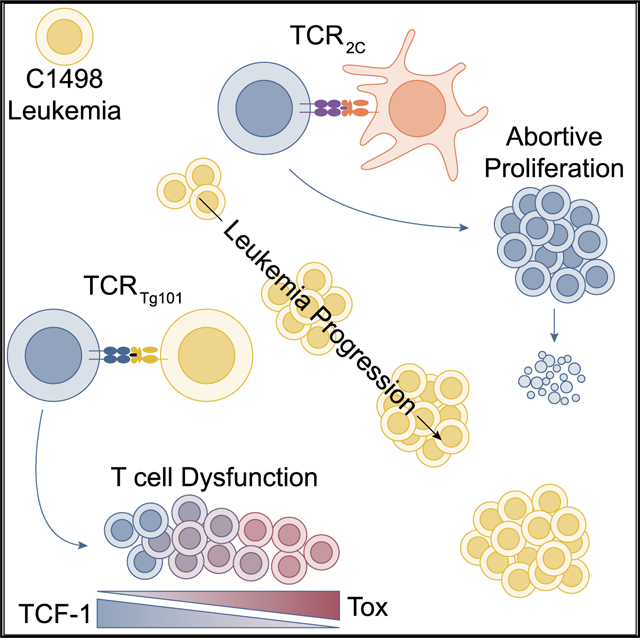

## INTRODUCTION

CD8^+^ T cells are key effectors of anti-tumor immune responses. In order to evade immune recognition and elimination, cancers exploit pathways that undermine CD8^+^ T cell responses, thereby facilitating cancer progression and metastasis (Gajewski et al., 2006). Although the existence of a dysfunctional CD8^+^ T cell phenotype has been demonstrated across many cancers, the underlying mechanisms and manifestations of T cell dysfunction are varied. Moreover, the degree to which tumor-specific CD8^+^ T cell fates are shaped by unique interactions with antigen-presenting cells (APCs) has not been well-defined.

Our group has been focused on characterizing immune evasion mechanisms in hosts with hematologic malignancies. We previously demonstrated in leukemia-bearing mice that cross-presentation of a leukemia-specific antigen by splenic CD8α^+^ type 1 conventional dendritic cells (cDC1s) induced the deletion of a CD8^+^ T cell population expressing a high-affinity T cell receptor (TCR; referred to as TCR_2C_) (Kline et al., 2018; Zhang et al., 2013). Interestingly, presentation of the antigen by cDC1s in draining lymph nodes (dLNs) of mice with locally implanted tumors derived from the same leukemia cells resulted in the robust activation of the identical CD8^+^ T cell population (Kline et al., 2018; Zhang et al., 2013), revealing that the environmental context in which a tumor antigen is encountered can confer drastically disparate CD8^+^ T cell functional states.

In order to elucidate the extent to which deletion was a fate shared by other leukemia-specific CD8^+^ T cells, we generated a TCR transgenic mouse strain (referred to as Tg101) harboring a clonal CD8^+^ T cell population specific for a naturally expressed, major histocompatibility complex (MHC) class I-restricted antigen on murine C1498 leukemia cells. In striking contrast to our previous observations (Zhang et al., 2013), CD8^+^ T cells from Tg101 mice (TCR_Tg101_) expanded to relatively large numbers in leukemia-bearing animals rather than being deleted. However, TCR_Tg101_ expansion in this context was accompanied by the progressive acquisition of an exhausted phenotype that could not be effectively reversed. Interestingly, the *in vivo* differentiation of TCR_Tg101_ into an exhausted state occurred independently of DCs but rather required direct presentation of cognate antigen by leukemia cells. These results reveal that multiple mechanisms mediate CD8^+^ T cell tolerance in leukemia and further highlight that the context in which a CD8^+^ T cell encounters its cognate leukemia antigen is a critical factor in determining the subsequent tolerance phenotype that ensues.

## RESULTS

### TCR_Tg101_ develop along the CD8 lineage and are leukemia specific

Tg101 transgenic mice were generated from TCR-α and -β chains of a CD8^+^ T cell clone (T15) specific for an undefined antigen expressed by C1498 leukemia cells (Boyer et al., 1997). Tg101 mice were born in Mendelian ratios, developed normally, and showed no gross symptoms or signs of poor health. Tg101 mice were crossed onto a *Rag2*^−/−^ background to prevent rearrangement of endogenous TCR-α and -β loci. Thymocytes from *Rag2*^−/−^ Tg101 mice developed into CD8 single-positive cells (Figures S1A–S1C), and TCR_Tg101_ were uniformly CD8^+^ in secondary lymphoid organs (SLOs) (Figures S1D–S1F). TCR_Tg101_ exhibited no evidence of self-reactivity and maintained a naive phenotype that persisted in SLOs of older mice (Figures S1G–S1K).

**Figure 1.**
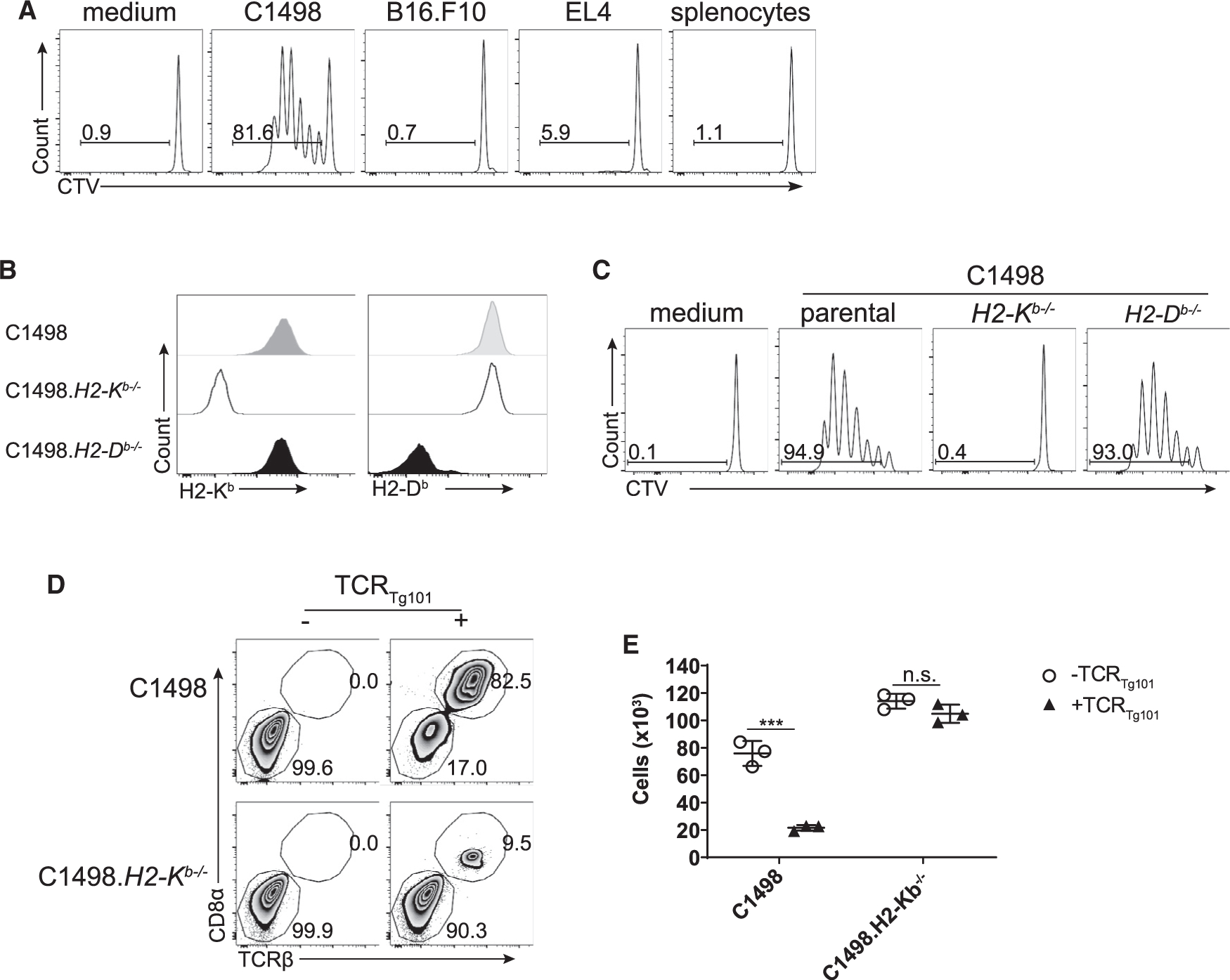
TCR_Tg101_ recognize an H2-K^b^-restricted antigen on C1498 cells (A) CTV-labeled TCR_Tg101_ were cultured for 72 h with the indicated tumor cell lines or with splenocytes from C57BL/6 mice. Proliferation of TCR_Tg101_, as measured by CTV dilution, was analyzed by flow cytometry. Representative FACS plots are shown. (B) Expression of H2-K^b^ and H2-D^b^ on parental C1498 cells, C1498.*H2*-*K*^*b*−/−^ cells, and C1498.*H2-Db*^−/−^ cells. Representative FACS plots are displayed. (C) Proliferation of CTV-labeled TCR_Tg101_ cultured for 72 h with C1498 cells, C1498.*H2*-*K*^*b*−/−^ cells, or C1498.*H2-Db*^−/−^ cells. Representative FACS plots are shown. (D and E) Live C1498 or C1498.*H2*-*K*^*b*−/−^ cells (2 × 10^4^) were cultured for 72 h alone or with 1 × 10^5^ TCR_Tg101_. Subsequently, numbers of viable C1498 or C1498.*H2*-*K*^*b*−/−^ cells were enumerated. Representative FACS plots are shown in (D). Summary data are presented in (E) as mean ± SD. Data are representative of 2 to 3 independent experiments with 3 wells/group. ***p < 0.001; n.s., not significant.

To confirm their specificity for syngeneic C1498 leukemia cells, CellTrace Violet (CTV)-labeled TCR_Tg101_ were cultured with C1498 cells or with other syngeneic cancer cell lines, including B16.F10 melanoma and EL4 thymoma, or with splenocytes from C57BL/6 mice. CTV dilution of TCR_Tg101_ occurred only upon co-culture with C1498 cells but not with B16.F10 cells, EL4 cells, or C57BL/6 splenocytes (Figure 1A), indicating that TCR_Tg101_ specifically recognize an antigen expressed on C1498 leukemia cells. Because TCR_Tg101_ failed to proliferate when co-cultured with syngeneic splenocytes and maintained a naive phenotype in Tg101 transgenic mice, it is unlikely that their cognate antigen is derived from a normal self-protein. Together, these results suggest that TCR_Tg101_ recognize a leukemia-specific antigen.

C1498 cells deficient in the MHC class I molecules H2-K^b^ (K^b^) or H2-D^b^ (D^b^) were generated in order to define the restricting MHC class I molecule for the Tg101 antigen (Figure 1B). Only *K*^*b*−/−^, but not parental or *D*^*b*−/−^ C1498, cells were incapable of stimulating proliferation of TCR_Tg101_ *in vitro*, demonstrating that TCR_Tg101_ recognize a K^b^-restricted leukemia antigen (Figure 1C). Importantly, parental C1498 cells, but not C1498 *K*^*b*−/−^ cells, were susceptible to TCR_Tg101_-mediated killing *in vitro* (Figures 1D and 1E). Thus, TCR_Tg101_ recognize a leukemia-specific antigen directly presented by C1498 leukemia cells and, in doing so, are capable of eliminating C1498 cells *in vitro*.

### TCR_Tg101_ accumulate and acquire an exhausted phenotype in leukemia-bearing hosts

**Figure 2.**
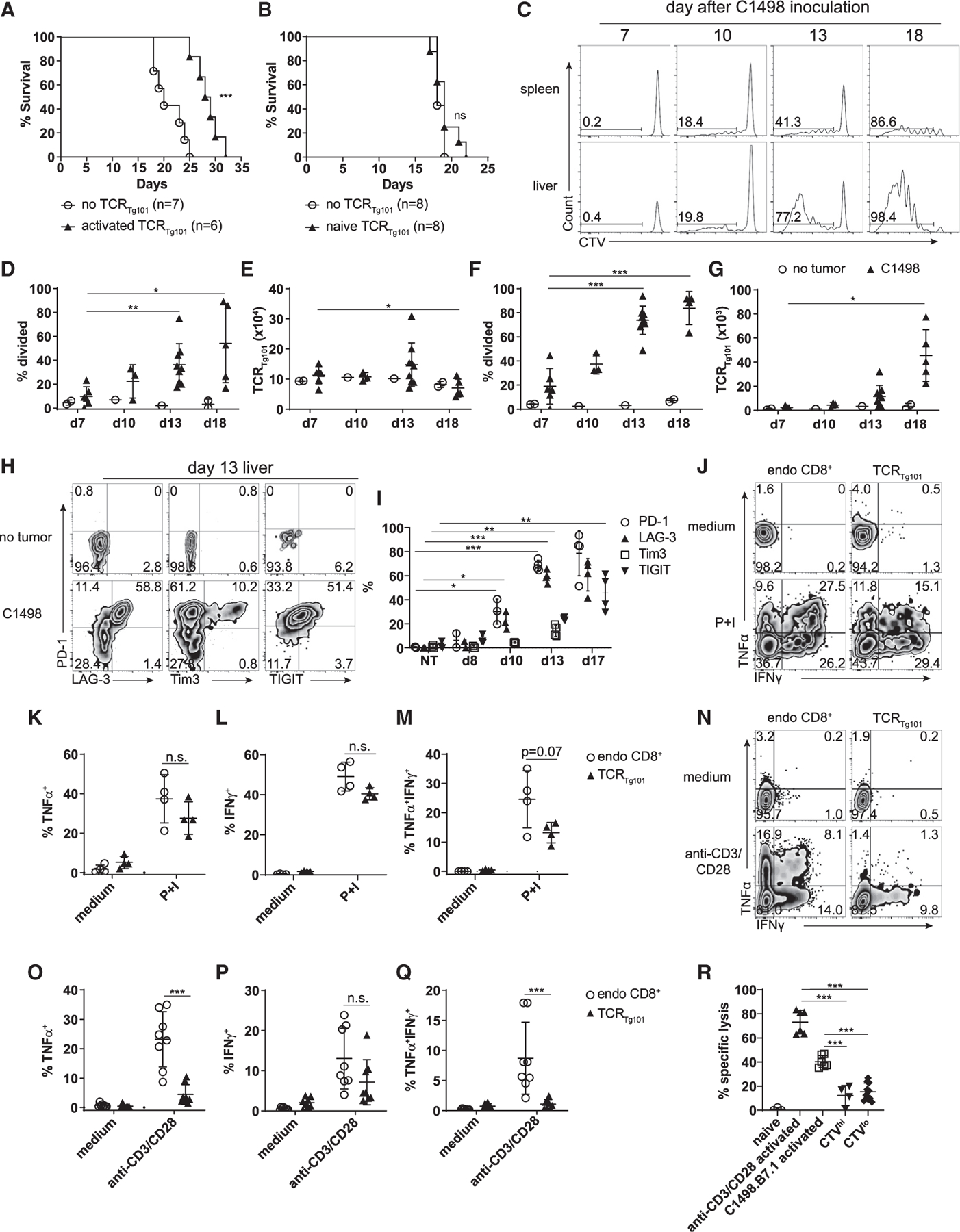
TCR_Tg101_ expand and acquire a dysfunctional phenotype in leukemia-bearing animals (A and B) Survival of C57BL/6 mice challenged i.v. with C1498 cells (10^6^) and transferred or not with in vitro activated (A) or naive (B) TCR_Tg101_ (4 × 10^6^) 3 days later. Data are pooled from 2 independent experiments with 3–4 mice/group. (C–G) 2 × 10^6^ CTV-labeled TCR_Tg101_ (CD45.2) were transferred into B6.SJL mice (CD45.1) followed by an i.v. challenge with 10^6^ C1498 leukemia cells 1 day later. (C) Representative FACS plots showing CTV dilution of adoptively transferred TCR_Tg101_ in spleens and livers of leukemia-bearing mice at the indicated time points. (D–G) Quantitative data showing the percentage of divided TCR_Tg101_ (D and F) and TCR_Tg101_ number (E and G) in spleens (D and E) or livers (F and G) of leukemia-bearing mice over time. Data are pooled from 2 to 3 independent experiments with 2 to 4 mice/group including data from leukemia-free control mice. (H) Representative FACS plots showing expression of PD-1, LAG-3, TIM3, and TIGIT on TCR_Tg101_ in livers of tumor-free or leukemia-bearing mice. (I) Quantitative data from (H) as mean ± SD. (J–Q) Mononuclear cells isolated from livers of mice 13 to 14 days (J–M) or 17 to 18 days (N–Q) after C1498 cell inoculation were restimulated with PMA + ionomycin (P+I) (J–M) or anti-CD3 and anti-CD28 antibodies (N–Q) for 4 h. Cytokine production was analyzed by flow cytometry. Gating was performed on TCRβ^+^CD8^+^CD45.1 cells (endogenous CD8^+^ T cells) or TCRβ^+^CD8^+^CD45.2 cells (TCR_Tg101_). (J and N) Representative FACS plots showing TNFα and IFNγ production by endogenous CD8^+^ T cells or TCR_Tg101_. Quantitative data are shown in (K)–(M) and (O)–(Q) as mean ± SD. (R) TCR_Tg101_ killing assay. CTV^hi^ and CTV^lo^ TCR_Tg101_ (CD45.1.2) were FACS-purified from livers of mice 17 to 18 days after C1498 inoculation and were co-cultured for 20 h with equal numbers of C1498 cells and EL4 cells (both CD45.2) labeled with different CTV concentrations. Naive TCR_Tg101_ purified from control Tg101 mice, or those activated *in vitro* with anti-CD3 and anti-CD28 antibodies or with C1498.B7.1 cells served as negative and positive controls for C1498 cell lysis, respectively. Specific lysis was analyzed by flow cytometry. Gating was performed on live CD8^−^CD45.2 cells. Data are representative (K–M) of or pooled (I and O–R) from 2 independent experiments with 2 to 4 mice/group and shown as mean ± SD. *p < 0.05; **p < 0.01; ***p < 0.001; n.s., not significant; NT, no tumor.

After confirming TCR_Tg101_ specificity for C1498 leukemia cells, their behavior in leukemia-bearing mice was characterized. Adoptive transfer of TCR_Tg101_ activated *in vitro* with anti-CD3 and anti-CD28 antibodies modestly extended the survival of C1498-challenged mice by approximately 8 to 9 days (Figure 2A). Conversely, adoptive transfer of large numbers of naive TCR_Tg101_ had no impact on the survival of leukemia-bearing mice (Figure 2B), which was suggestive of an acquired tolerant state among TCR_Tg101_ *in vivo*. For comparison, adoptive transfer of naive or *in vitro*-activated TCR_2C_ failed to extend the survival of leukemia-bearing mice (Figures S2A and S2B). To further investigate the hypothesis that TCR_Tg101_ acquired a dysfunctional phenotype in leukemia-bearing hosts, the fate of naive CTV-labeled TCR_Tg101_ was assessed following adoptive transfer into C57BL/6 hosts that received an intravenous (i.v.) challenge with C1498 cells the following day. 7 days later, TCR_Tg101_ remained largely undivided and had not upregulated CD44 expression in the spleen, where we have previously demonstrated leukemia antigen cross-presentation occurs (Kline et al., 2018). In the liver, which is a primary site of leukemia progression in the C1498 model (Zhang et al., 2009), very few TCR_Tg101_ had proliferated, although a subset began to express CD44 (Figures 2C–2G), indicating that most TCR_Tg101_ had not yet encountered cognate antigens. Expression of co-inhibitory receptors (PD-1, LAG-3, TIM3, TIGIT) on TCR_Tg101_ was negligible at this time point (Figure 2I). By day 13, a fraction of TCR_Tg101_ had begun to proliferate and upregulate CD44 expression (Figures 2C–2G), and a subset expressed PD-1 and LAG-3 (Figures 2H and 2I). Finally, at days 17 to 18, TCR_Tg101_ had proliferated extensively, particularly in livers of leukemia-bearing animals (Figures 2C–2G). At this late time point, the majority of TCR_Tg101_ were CD44^+^, PD-1^+^, and LAG-3^+^, and many co-expressed TIM3 and TIGIT (Figure 2I). Between days 7 and 18, TCR_Tg101_ expanded up to 16-fold in livers of leukemia-bearing mice (Figure 2G). The expansion and upregulation of co-inhibitory receptors by TCR_Tg101_ is in contrast to previous observations with TCR_2C_, which were rapidly and efficiently deleted in mice with leukemia (Kline et al., 2018; Zhang et al., 2013), and is consistent with the acquisition of a dysfunctional phenotype.

To directly assess the functional capacity of TCR_Tg101_ in mice with advanced leukemia, mononuclear cells were isolated from livers of leukemia-bearing mice 14 and 18 days following TCR_Tg101_ adoptive transfer (13 to 17 days following C1498 cell challenge) and were restimulated *ex vivo* with anti-CD3 and anti-CD28 antibodies or phorbol 12-myristate 13-acetate (PMA) and ionomycin. TCR_Tg101_ produced non-significantly lower levels of tumor necrosis factor (TNF)α and interferon (IFN)γ (or both) than endogenous CD8^+^ T cells at day 14 (Figure 2J–2M). At day 18, effector cytokine production by TCR_Tg101_ had further declined, and when compared with endogenous CD8^+^ T cells, TCR_Tg101_ expressed significantly lower levels of TNFα and tended to also be poorer producers of IFNγ (Figures 2N–2P). Importantly, a significantly smaller proportion of TCR_Tg101_ produced both effector cytokines at this time point (Figure 2Q). Lack of polyfunctional cytokine production has been recurrently observed in dysfunctional tumor-infiltrating CD8^+^ T cells (Ahmadzadeh et al., 2009; Sakuishi et al., 2010; Thommen and Schumacher, 2018). Granzyme B (GzmB) expression induced by *ex vivo* restimulation with anti-CD3 and anti-CD28 antibodies was similar in liver-infiltrating TCR_Tg101_ and endogenous CD8^+^ T cells (Figures S3A–S3C). Interestingly, *ex vivo* restimulation of TCR_Tg101_ with C1498 cells failed to activate effector cytokine production or GzmB expression. However, restimulation of TCR_Tg101_ with C1498 cells engineered to express the T cell co-stimulatory molecule B7.1 (C1498.B7.1) induced cytokine production and GzmB expression, albeit to a lesser degree than anti-CD3 and anti-CD28 antibodies (Figures S3A–S3C).

To determine the extent to which extrinsic inhibitory signals delivered by C1498 cells or other immune cells present after Ficoll-based enrichment of liver-resident mononuclear cells were extrinsically mediating the poor effector function of *ex vivo* restimulated TCR_Tg101_, liver-resident CD8^+^ T cells (including TCR_Tg101_) were purified via positive selection at day 18 following C1498 challenge. The function of purified or bulk endogenous CD8^+^ T cells and TCR_Tg101_ was examined following *ex vivo* restimulation with anti-CD3 and anti-CD28 antibodies. TNFα production by purified endogenous CD8^+^ T cells and TCR_Tg101_ was slightly higher than the same T cell populations restimulated in the presence of contaminating immune cells and C1498 cells (bulk) while IFNγ production was unaffected (Figures S3D–S3F), suggesting that the acquired dysfunction of TCR_Tg101_ was largely cell intrinsic.

To directly examine the cytolytic capability of TCR_Tg101_ in leukemia-bearing animals, CTV^hi^ (naive/undivided) and CTV^lo^ (dysfunctional) TCR_Tg101_ (CD45.1.2) were separately fluorescence-activated cell sorting (FACS)-purified from livers of leukemia-bearing mice 18 days after adoptive transfer (see Figure 4A) and cultured with equal numbers of C1498 cells and EL4 cells (both CD45.2) labeled with 2 different CTV concentrations. As a positive control for C1498 cell cytolysis, TCR_Tg101_ isolated from naive Tg101 mice were restimulated *in vitro* with anti-CD3 and anti-CD28 antibodies or with gamma-irradiated C1498.B7.1 cells for 3 days prior to co-culture with CTV-labeled C1498 cells and EL4 cells. As a negative control for C1498 cell cytolysis, unstimulated TCR_Tg101_ freshly isolated from naive Tg101 mice were utilized. Specific lysis was measured by comparing relative proportions of viable C1498 and EL4 cells after a 20-hour co-culture with the indicated TCR_Tg101_ populations, compared with those present in culture wells lacking TCR_Tg101_. As expected, unstimulated TCR_Tg101_ from control Tg101 mice failed to eliminate C1498 cells, while *in vitro* anti-CD3 and anti-CD28 antibody-stimulated and C1498.B7.1-stimulated TCR_Tg101_ from control Tg101 mice effectively and specifically killed C1498 cells (Figure 2R). CTV^hi^ TCR_Tg101_ from livers of leukemia-bearing mice poorly lysed C1498 cells, which was not surprising given that they were likely of a naive phenotype. Finally, CTV^lo^ TCR_Tg101_ from livers of leukemia-bearing animals were also unable to effectively kill C1498 cells directly *ex vivo* (Figure 2R), despite their inducible expression of GzmB (Figures S3A and S3C). Collectively, these observations indicated that as TCR_Tg101_ expanded in leukemia-bearing animals, they concomitantly acquired a dysfunctional phenotype.

Although TCR_Tg101_ exhibited clear evidence of functional impairment, particularly at late stages of the disease, they largely retained proliferative capacity. Only TCR_Tg101_, having undergone greater than 6 to 7 cell divisions, showed decreased BrdU incorporation (Figures S3G and S3H). Furthermore, while a small subset of TCR_Tg101_ expressed activated caspase 3, most were viable, suggesting that the acquisition of effector dysfunction was not necessarily associated with the induction of apoptosis (Figures S3I and S3J). These results demonstrate that proliferative capacity and effector function were largely uncoupled in TCR_Tg101_ until the point at which the dysfunctional program was fully established—an observation previously described among antigen-specific CD8^+^ T cells in tumors (Schietinger et al., 2016).

### Dysfunctional TCR_Tg101_ acquire a transcriptional program canonically associated with T cell exhaustion

**Figure 3.**
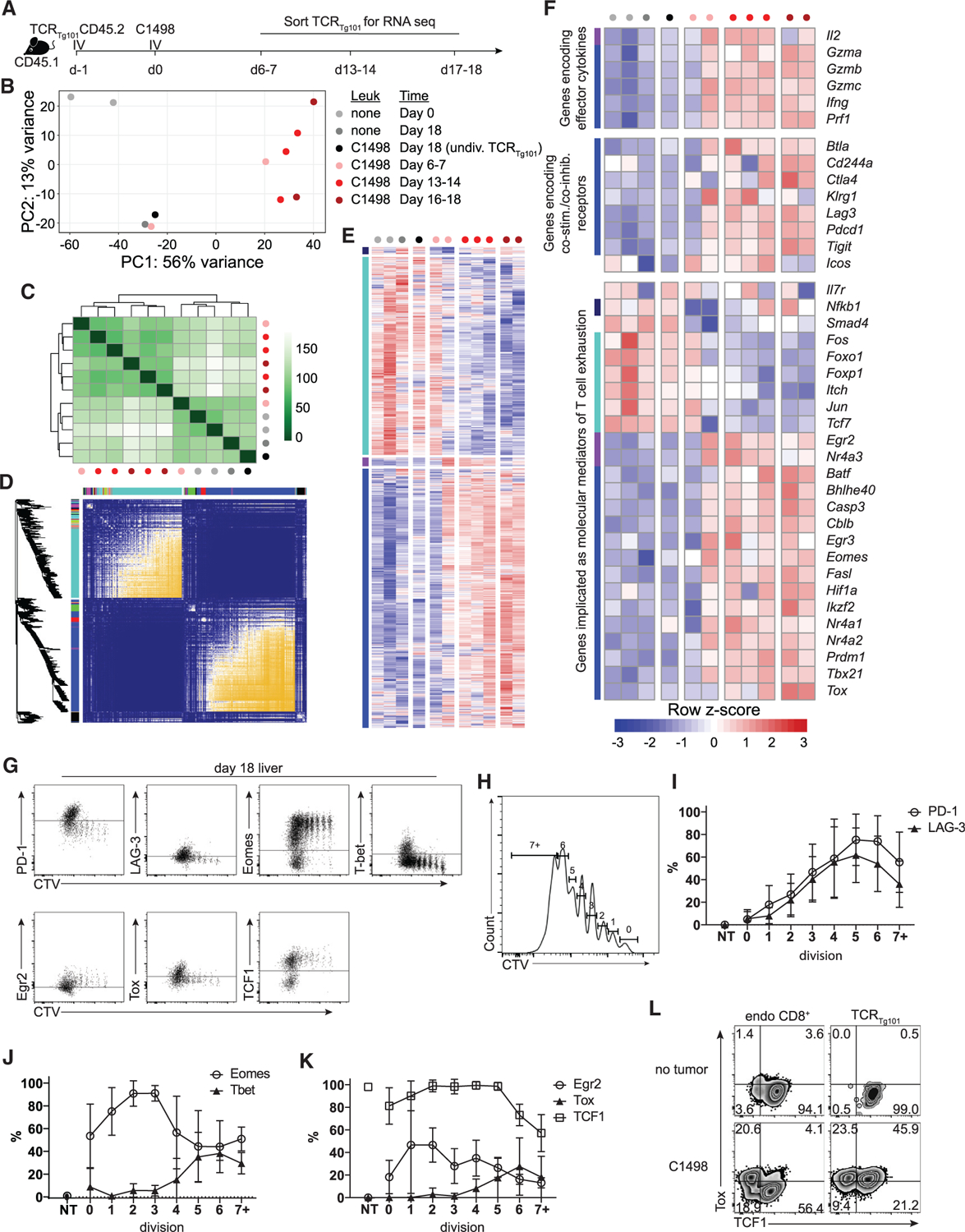
TCR_Tg101_ acquire a transcriptional program enriched for genes associated with T cell anergy and exhaustion (A) Experimental design. (B) Principal-component analysis (PCA). (C) Complete clustering of TCR_Tg101_ samples at the indicated time points based on their Euclidean distance. (D) Heatmap of topological overlap based on weighted gene correlation network analysis of TCR_Tg101_ transcriptomes. (E) Heatmap showing up/downregulation or transient up/downregulation of genes from 2 major and 2 minor nodules, respectively. (F) Heatmap showing up/downregulation of selected genes of interest (co-inhibitory receptors, transcription factors, effector cytokines, cell-cycle genes, survival genes) in TCR_Tg101_ isolated from leukemia-bearing mice at indicated time points. (G–L) PD-1, LAG-3, Eomes, T-bet, Egr2, Tox, and TCF1 expression in CTV-labeled TCR_Tg101_ from livers of leukemia-bearing mice at day 18. (G) Representative FACS plots showing expression of the indicated proteins by CTV dilution (cell division) in TCR_Tg101_. (H) Gating strategy for (I)–(K). (I–K) Quantitative data showing expression of PD-1 and LAG-3 (I), Eomes and T-bet (J), and Egr2, Tox, and TCF1 (K) in TCR_Tg101_ according to cell division number. Data are presented as mean ± SD (L) Representative FACS plots showing expression of Tox and TCF1 in liver-resident endogenous CD8^+^ T cells TCR_Tg101_.

To identify molecular programs associated with the observed dysfunctional TCR_Tg101_ phenotype, RNA sequencing (RNA-seq) was performed on TCR_Tg101_ isolated from livers of leukemia-bearing mice at several time points following adoptive transfer (days 6 to 7, 13 to 14, and 16 to 18) and on TCR_Tg101_ from control (leukemia-free) mice (Figure 3A). Principal-component analysis (PCA) indicated that TCR_Tg101_ from control animals, and those from leukemia-bearing animals at mid (days 13 to 14) to late (day 16 to 18) time points, exhibited very different transcriptional programs (Figure 3B). Complete clustering of the samples based on Euclidean distance revealed 2 distinct TCR_Tg101_ clusters, including one containing TCR_Tg101_ from control mice, and the other containing TCR_Tg101_ isolated from leukemia-bearing mice at mid and late time points (Figure 3C). Interestingly, one early (days 6 to 7) time point TCR_Tg101_ sample clustered with TCR_Tg101_ from control mice, while the other day-6-to-7 TCR_Tg101_ sample clustered with day-13-to-14 TCR_Tg101_ from leukemia-bearing mice (Figures 3B and 3C), likely indicating that TCR_Tg101_ had already encountered leukemia antigen in one sample but not the other. In order to identify differentially expressed genes by TCR_Tg101_ at specific time points during the course of leukemia progression, we performed pairwise comparisons of gene expression profiles at the time points indicated above (Figure S4). In total, 4,075 genes were significantly differentially expressed across all pairwise comparisons (Table S1).

To identify gene modules potentially involved in the regulation of TCR_Tg101_ dysfunction, we performed a weighted gene correlation network analysis (Langfelder and Horvath, 2008), an unbiased analysis of gene co-regulation previously utilized to define transcriptional networks associated with CD8^+^ T cell exhaustion in the context of chronic viral infection (Doering et al., 2012). Here, 2 major gene modules were observed—one defined by gene transcripts that were progressively upregulated (indicated in blue in Figures 3D and 3E) and another characterized by gene transcripts that were progressively downregulated over the course of the experiment (indicated in turquoise). 2 minor gene clusters containing genes that were transiently up- or downregulated (purple and navy, respectively) in TCR_Tg101_ early in leukemia-bearing mice were also identified (Figures 3D and 3E). Within the module that contained significantly upregulated genes were several encoding co-inhibitory receptors, including *Pdcd1, Tigit, Lag3,* and *Ctla4* (Figure 3F). Somewhat surprisingly, genes also contained within this cluster were those encoding effector cytokines (*Ifng*) and cytolytic molecules (*Gzma, Gzmb, Gzmc, Prf1*). Finally, within this cluster were genes encoding transcription factors previously associated with T cell exhaustion or anergy, including *Tox, Nr4a* family members, *Egr3*, and *Cblb* (Chen et al., 2019a; Doering et al., 2012; Jadhav et al., 2019; McLane et al., 2019; Philip et al., 2017; Seo et al., 2019; Sowell and Kaech, 2016). Conversely, within the module containing significantly downregulated genes were those encoding for transcription factors such as *Tcf7* and *Foxo1*, which antagonize terminal differentiation into a dysfunctional state (Figure 3F) (Chen et al., 2019b; Delpoux et al., 2018; Sade-Feldman et al., 2018; Siddiqui et al., 2019). Although these gene expression patterns suggest that TCR_Tg101_ acquire an exhausted phenotype in leukemia-bearing mice, they could also be consistent with the induction of CD8^+^ T cell anergy, as significant overlap exists among gene sets associated with various dysfunctional T cell states (Waugh et al., 2016). Together, these results implied that the hyporesponsive TCR_Tg101_ phenotype was coupled with transcriptional changes previously associated with T cell exhaustion/dysfunction.

Direct analysis of co-inhibitory receptor and transcription factor protein expression in TCR_Tg101_ from livers of late-stage, leukemia-bearing mice was next performed to validate key RNA-seq findings. Consistent with previous observations (Figures 2H and 2I) and RNA-seq analysis, PD-1 and LAG-3 were upregulated on TCR_Tg101_ in a manner that directly correlated with *in vivo* proliferation (Figures 3G–3I). Furthermore, Eomes was highly upregulated in TCR_Tg101_ upon antigen encounter and during early rounds of proliferation but was subsequently downregulated with continued TCR_Tg101_ expansion (Figures 3G and 3J). A similar but delayed pattern was observed for TCF1, a transcription factor associated with “stem-like” properties and responsiveness to PD-1 blockade therapy among CD8^+^ tumor-infiltrating lymphocytes (Sade-Feldman et al., 2018; Siddiqui et al., 2019). TCF1 expression was high in naive TCR_Tg101_ and those undergoing early proliferation but decreased significantly in TCR_Tg101_ that had proliferated extensively (Figures 3G and 3K). Conversely, transcription factors associated with CD8^+^ T cell dysfunction/exhaustion, including Egr2 and Tox (Scott et al., 2019; Zheng et al., 2012), were upregulated in TCR_Tg101_ following antigen encounter. Egr2 expression showed an earlier peak, whereas Tox expression was upregulated at a point where most TCR_Tg101_ had undergone 4 or more divisions (Figures 3G and 3K). Endogenous CD8^+^ T cells in livers of leukemia-bearing mice showed 2 clear populations of Tox and TCF1 expression (TCF1^+^Tox^−^ and TCF1^−^Tox^+^), suggesting that TCF1 downregulation was associated with Tox expression, as might be expected (Figure 3L). Conversely, among liver-resident TCR_Tg101_, downregulation of TCF1 was not required for Tox upregulation (Figure 3L), which has previously been reported among dysfunctional, antigen-specific CD8^+^ T cells (Sekine et al., 2020). Thus, TCR_Tg101_ progressively downregulated expression of transcription factors associated with plasticity and differentiation into effector and memory subsets and upregulated expression of transcription factors known to drive an exhaustion program in dysfunctional CD8^+^ T cells, both in tumors and chronic viral infections.

### The dysfunctional TCR_Tg101_ phenotype is T cell intrinsic and irreversible

**Figure 4.**
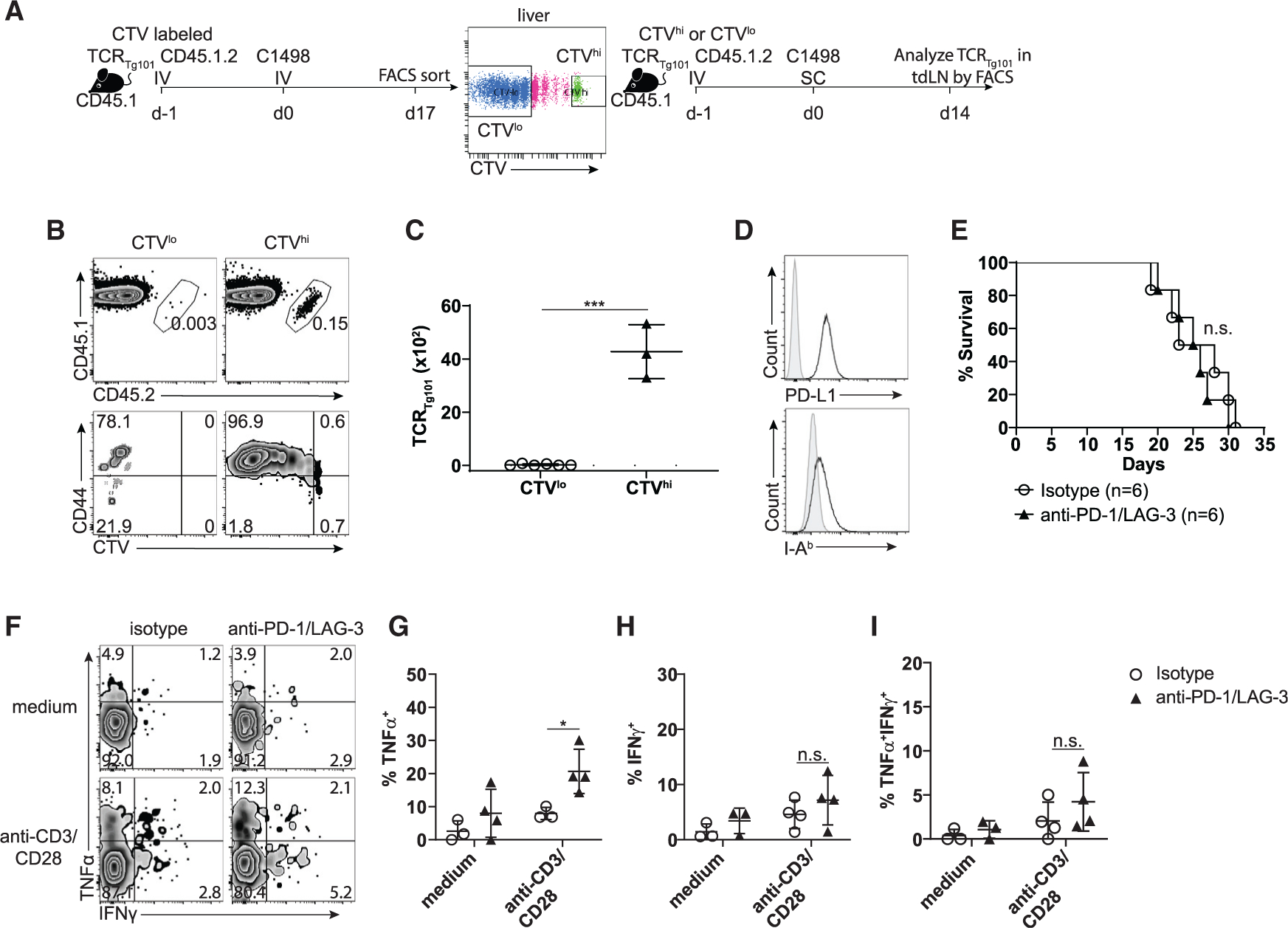
The exhausted TCR_Tg101_ phenotype is profound (A) Experimental design: 8 × 10^6^ CTV-labeled TCR_Tg101_ (CD45.1.2) were transferred into B6.SJL (CD45.1) mice followed by i.v. challenge with C1498 leukemia cells 1 day later. Naive (CTV^hi^) or exhausted (CTV^lo^) TCR_Tg101_ were isolated from livers of day-17 leukemia-bearing mice by FACS and were separately transferred into secondary B6.SJL hosts. These B6.SJL mice received a s.c. C1498 cell (10^6^) challenge the following day. On day 14, numbers and CD44 expression of TCR_Tg101_ in tdLNs were analyzed by flow cytometry. (B) Representative FACS plots showing CTV dilution and CD44 expression of TCR_Tg101_ in tdLNs of secondary B6.SJL mice with s.c. C1498 tumors. (C) Quantified data showing numbers of TCR_Tg101_ in tdLNs of secondary B6.SJL mice with s.c. C1498 tumors. (D) Expression of PD-L1 and I-A^b^ on C1498 cells when analyzed directly ex vivo. Shaded histograms represent staining with appropriate isotype control antibodies. (E) TCR_Tg101_ (8 × 10^6^) were adoptively transferred into C57BL/6 mice. 1 day later, mice were challenged i.v. with C1498 cells (10^6^) and were treated with anti-PD-1 and anti-LAG-3 or isotype control antibodies. Survival was assessed. (F–I) Cytokine production by TCR_Tg101_ in leukemia-bearing mice treated with 4 doses of isotype control or anti-PD-1/anti-LAG-3 antibody therapy. Mononuclear cells isolated from livers on day 16 were restimulated *ex vivo* with anti-CD3 and anti-CD28 antibodies and cytokine production was assessed. Gating was performed on TCRβ^+^CD8^+^CD45.1.2 cells (TCR_Tg101_). (F) Representative FACS plots showing TNFα and IFNγ production by TCR_Tg101_. Quantitative data are shown in (G)–(I) as mean ± SD. Data are pooled from 2 independent experiments with 2 to 3 mice/group. *p < 0.05; ***p < 0.001; n.s., not significant.

Having clearly established that TCR_Tg101_ exhibit a dysfunctional phenotype in leukemia-bearing animals, we sought to determine the extent to which TCR_Tg101_ function could be rescued by removing them from the leukemia environment and introducing them into secondary recipients that subsequently received a subcutaneous (s.c.) C1498 cell challenge (Figure 4A). Thus, CTV-labeled TCR_Tg101_ were isolated from livers of day-17 leukemia-bearing mice. Non-proliferating TCR_Tg101_ (naive; CTV^hi^) and TCR_Tg101_, having undergone >4 rounds of cell division (exhausted; CTV^lo^), were separately isolated and adoptively transferred into secondary recipients. 1 day later, secondary hosts were challenged with C1498 cells s.c. On day 14, numbers and proliferation of TCR_Tg101_ were assessed in tumor-dLNs (tdLNs) (Figure 4A). Naive (CTV^hi^) TCR_Tg101_ expanded vigorously and upregulated CD44 in tdLNs of secondary recipients, as expected (Figures 4B and 4C). In contrast, exhausted (CTV^lo^) TCR_Tg101_ were almost completely undetectable in tdLNs or within C1498 tumors, spleens, or other selected organs of secondary hosts (Figures 4B, 4C, and S5A). To determine if resting dysfunctional (CTV^lo^) TCR_Tg101_ *in vitro* with cytokine support could restore their ability to survive and expand, CTV^hi^ and CTV^lo^ TCR_Tg101_ isolated from livers of leukemia-bearing animals were cultured with interleukin (IL)-2, IL-7, and IL-15 for 5 days, at which point, CTV^hi^ TCR_Tg101_ had expanded 1.5-fold; conversely, very few CTV^lo^ TCR_Tg101_ survived (Figures S5B and S5C). Finally, to determine whether optimal *ex vivo* stimulation through the TCR, CD28, and provision of IL-12 could rescue the survival and function of dysfunctional TCR_Tg101_, CTV^hi^ and CTV^lo^ TCR_Tg101_ were purified from livers of day-17 leukemia-bearing mice and restimulated *ex vivo* with anti-CD3 and anti-CD28 antibodies or with C1498.B7.1 cells along with IL-2 and IL-12 for 5 days. As shown in Figure S5D, CTV^hi^ TCR_Tg101_ expanded markedly upon restimulation with anti-CD3 and anti-CD28 antibodies or with C1498.B7.1 cells plus IL-2 and IL-12, while again, very few CTV^lo^ TCR_Tg101_ survived. Furthermore, anti-CD3 and anti-CD28 antibodies plus IL-2/IL-12 restimulation of CTV^hi^ TCR_Tg101_ induced robust production of TNFα and IFNγ and expression of GzmB. Restimulation of CTV^hi^ TCR_Tg101_ with C1498.B7.1 cells plus IL-2/IL-12 induced uniform GzmB expression, while production of IFNγ and TNFα was less impressive (Figures S5E–S5L). With regard to CTV^lo^ TCR_Tg101_, restimulation with anti-CD3 and anti-CD28 antibodies plus IL-2/IL-12 induced partial GzmB expression and IFNγ/TNFα production among the few surviving cells, whereas absolute numbers of effector cytokine-producing and GzmB-expressing CTV^lo^ TCR_Tg101_ were strikingly decreased when compared to their CTV^hi^ TCR_Tg101_ counterparts (Figures S5E–S5L). Taken together, these results suggest that TCR_Tg101_ exhaustion was intrinsic and not readily reversible upon removal from the leukemia environment.

The dysfunctional TCR_Tg101_ phenotype was temporally associated with upregulation of multiple co-inhibitory receptors, primarily PD-1 and LAG-3 (Figures 2H and 2I). To determine whether interruption of PD-1/PD-L1 and LAG-3/MHC class II interactions could rescue the function of exhausted TCR_Tg101_, mice transferred with TCR_Tg101_ and challenged i.v. with C1498 cells were treated with anti-PD-1 and anti-LAG-3 antibodies. C1498 cells expressed both PD-L1 and low levels of MHC class II molecules (I-A^b^), the ligands for PD-1 and LAG-3, respectively, when analyzed *ex vivo* (Figure 4D). Despite these observations, treatment with anti-PD-1 and anti-LAG-3 antibodies failed to enhance the survival of leukemia-bearing mice transferred with TCR_Tg101_ (Figure 4E) and did little to reverse the dysfunctional TCR_Tg101_ phenotype (Figures 4F–4I). These results indicated that either co-inhibitory receptor expression was dispensable in promoting the exhausted TCR_Tg101_ phenotype, or, more likely, that the exhaustion program evolved to become independent of key T cell checkpoint receptors.

### *In vivo* antigen recognition by TCR_Tg101_ requires direct presentation by leukemia cells

**Figure 5.**
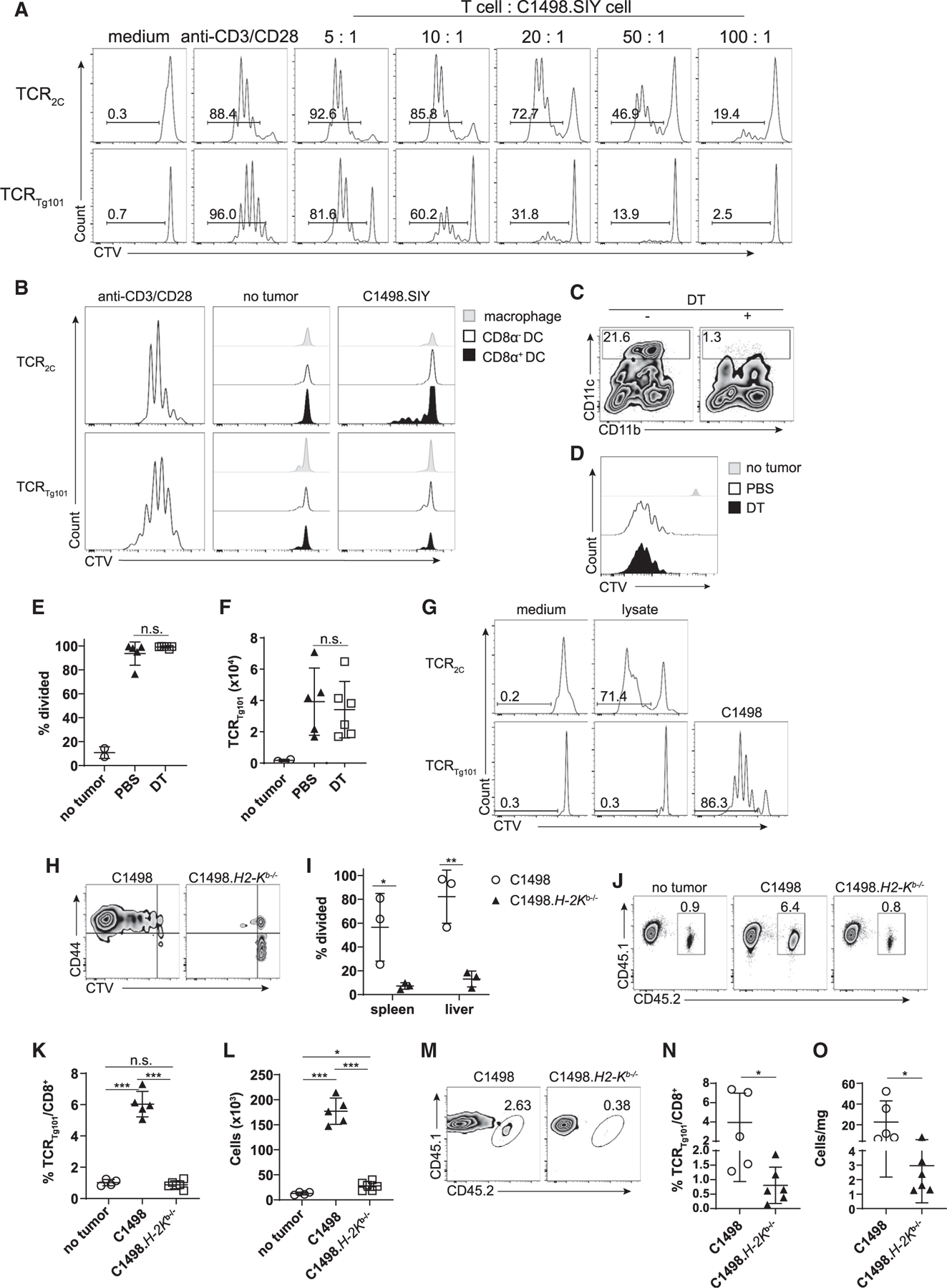
Direct antigen presentation by leukemia cells is required for *in vivo* antigen recognition by TCR_Tg101_ (A) TCR_2C_ or TCR_Tg101_ (1 × 10^5^) were CTV-labeled and cultured *in vitro* with C1498.SIY cells at various ratios for 72 h, at which point TCR_2C_ and TCR_Tg101_ proliferation, as measured by CTV dilution, was assessed by flow cytometry. TCR_2C_ and TCR_Tg101_ stimulated with anti-CD3 and anti-CD28 antibodies or left unstimulated served as positive and negative controls for proliferation, respectively. (B) CTV dilution profiles of TCR_2C_ or TCR_Tg101_ cultured for 72 h with CD8α^+^ DCs, CD8 α^−^ DCs, or macrophages isolated from spleens of naive C57BL/6 mice or C57BL/6 mice challenged 3 h earlier with C1498.SIY cells i.v. (C–F) CTV-labeled TCR_Tg101_ (CD45.1.2) were adoptively transferred into CD11c-DTR bone marrow chimeric mice (CD45.2) that received DT or PBS prior to and following i.v. C1498 cell challenge. (C) Representative FACS plot showing frequencies of CD11c^+^ cells in spleens of CD11c-DTR bone marrow chimeras after DT or PBS treatment. Gating was performed on live CD3^−^CD19^−^CD11c^+^ cells. (D) Representative FACS plots showing CTV dilution of TCR_Tg101_ (TCRb^+^CD8^+^CD45.1.2 cells) in livers of naive or C1498 cell-challenged CD11c-DTR bone marrow chimeric mice (treated with DT or PBS) at day 12. (E and F) Quantitative data from (D) are shown. (G) CTV dilution profiles of TCR_2C_ and TCR_Tg101_ cultured with BMDCs pulsed with C1498.SIY or C1498 cell lysates, respectively. CTV dilution profile of TCR_Tg101_ cultured with live C1498 cells was included as a positive control (lower right). Representative FACS plots are shown. (H and I) 2 × 10^6^ CTV-labeled TCR_Tg101_ (CD45.2) were transferred i.v. into B6.SJL (CD45.1) mice followed by an i.v. challenge with 10^6^ C1498 or C1498.H2-Kb^−/−^ cells the following day. On day 18, TCR_Tg101_ proliferation was assessed. (H) Representative FACS plots showing CTV dilution and CD44 expression on TCR_Tg101_ in livers of mice previously challenged with C1498 or C1498.H2-Kb^−/−^ cells. (I) Quantitative data from (H) are shown. (J–L) 2 × 10^6^ CTV-labeled TCR_Tg101_ (CD45.1.2) were transferred i.v. into B6.SJL (CD45.1) mice followed by a s.c. challenge with 10^6^ C1498 or C1498.H2-Kb^−/−^ cells the following day. 14 days later, TCR_Tg101_ frequencies and numbers were assessed in tdLNs and tumors. (J) Representative FACS plots showing TCR_Tg101_ frequencies in tdLNs of mice with s.c. C1498 or Kb^−/−^ C1498 tumors. (K and L) Quantitative data showing frequencies (K) and numbers (L) of TCR_Tg101_ in tdLNs of mice with s.c C1498 or Kb^−/−^ C1498 tumors. (M) Representative FACS plots showing TCR_Tg101_ frequencies among total CD8^+^ T cell populations in s.c. C1498 or Kb^−/−^ C1498 tumors. (N and O) Quantitative data showing TCR_Tg101_ frequencies (N) and numbers/mg of tumor tissue (O). Data are representative (I) of or pooled (E–F, K–L, and M–O) from 2 independent experiments with 3 mice/group and presented as mean ± SD. *p < 0.05; **p < 0.01; ***p < 0.001; n.s., not significant.

We previously showed that leukemia antigen recognition by and deletion of TCR_2C_ was mediated by antigen cross-presenting splenic CD8α^+^ cDC1s (Kline et al., 2018). Moreover, in leukemia-bearing *Batf3*^−/−^ mice largely devoid of cDC1s (Hildner et al., 2008), TCR_2C_ maintained a naive phenotype and failed to proliferate (Kline et al., 2018). Despite their near-complete inability to engage cognate antigen following C1498.SIY leukemia cell challenge in the absence of cross-presenting cDC1s, TCR_2C_ clearly recognized antigens and proliferated following direct presentation by C1498.SIY cells *in vitro*, possibly with higher sensitivity than TCR_Tg101_ (Figure 5A). However, without knowing the actual affinity of TCR_Tg101_ for cognate antigens or the relative extent to which the antigens recognized by TCR_2C_ versus TCR_Tg101_ are expressed on C1498 cells, this conclusion is speculative. To determine if cross-presentation by cDC1s was similarly required for *in vivo* recognition of the Tg101 antigen, TCR_Tg101_ were transferred into *Batf3*^−/−^ or *Batf3*^+/+^ mice that were challenged i.v. with C1498 cells the following day. Numbers and proliferation of TCR_Tg101_ were similar in leukemia-bearing *Batf3*^−/−^ and *Batf3*^+/+^ mice, indicating that cDC1s—the major subset of cross-presenting DCs—were not required for antigen recognition and expansion of TCR_Tg101_ *in vivo* (Figures S6A–S6F).

Cross-presentation is a major pathway by which tumor antigens are displayed to CD8^+^ T cells (Hildner et al., 2008; Theisen et al., 2018). The observation that cDC1s were dispensable for TCR_Tg101_ expansion in leukemia-bearing animals suggested either that the Tg101 antigen was cross-presented by a separate APC population or that a different antigen-presentation pathway was involved altogether. To address the first possibility, CD8α^+^ cDC1, CD11b^+^ cDC2, and CD11b^+^CD11c^lo/−^ cells (monocytes/macrophages) were isolated from spleens of mice challenged i.v. 3 h earlier with C1498 cells expressing the K^b^-restricted SIYRYYGL peptide antigen (C1498.SIY) recognized by TCR_2C_. We have demonstrated that a fraction of splenic CD8α^+^ cDC1s engulf C1498 cell-derived material and cross-present the SIY antigen to TCR_2C_ at this time point (Kline et al., 2018). Consistent with previous results, CD8 α^+^ cDC1s, but not CD11b^+^ cDC2s or macrophages/monocytes, induced proliferation of TCR_2C_ directly *ex vivo*, consistent with their ability to cross-present the SIY antigen. However, none of these APC populations supported TCR_Tg101_ activation *ex vivo* (Figure 5B), arguing that the Tg101 antigen was not effectively cross-presented, at least at the time point assessed in this experiment.

To determine whether DCs were at all capable of presenting the Tg101 antigen *in vivo*, *Itgax*^*DTR-EGFP*^ (CD11c-DTR) bone marrow chimeric mice were generated. 8 weeks later, CD11c-DTR bone marrow chimeric animals received adoptive transfer of TCR_Tg101_ followed 1 day later by i.v. C1498 cell challenge. CD11c-DTR chimeras also received DT or PBS 1 day prior to TCR_Tg101_ transfer and every 2 days thereafter for a total of 7 doses. As shown in Figure 5C, DT effectively depleted CD11c^+^ cells from CD11c-DTR bone marrow chimeric animals. Surprisingly, TCR_Tg101_ numbers and proliferation profiles were identical in the presence or absence of CD11c^+^ cells (Figures 5D–5F), indicating that DCs are dispensable for *in vivo* antigen presentation to TCR_Tg101_. Lastly, bone-marrow-derived DCs (BMDCs) pulsed with C1498 cell lysates were also incapable of inducing TCR_Tg101_ proliferation *in vitro* (Figure 5G), consistent with our *in vivo* findings. Collectively, these observations revealed that the Tg101 antigen was not efficiently displayed through cross-presentation and suggested that an alternative presentation pathway mediated antigen recognition by TCR_Tg101_ *in vivo*.

To determine whether direct Tg101 antigen presentation by C1498 leukemia cells was required for *in vivo* antigen encounter by TCR_Tg101_, their expansion was compared in mice challenged i.v. with parental C1498 or C1498 *K*^*b*−/−^ cells. TCR_Tg101_ expanded in mice challenged with parental C1498 cells as expected. However, no TCR_Tg101_ expansion occurred in mice inoculated with C1498 *K*^*b*−/−^ cells (Figures 5H and 5I). A caveat of this experiment was that the survival of mice challenged with C1498 *K*^*b*−/−^ cells was significantly prolonged compared to mice challenged with parental C1498 cells. This difference persisted in C57BL/6 mice, in which natural killer (NK) cells were depleted, and in *Rag*^−/−^γ_*c*_^−/−^ mice, which lacked T cells, B cells, and NK cells. These results suggested that engraftment and/or progression of C1498 *K*^*b*−/−^ leukemia was impaired, potentially through enhanced phagocytosis of leukemia cells with diminished MHC class I expression. Fortunately, parental C1498 and C1498 *K*^*b*−/−^ tumors grew similarly following s.c. inoculation in C57BL/6 mice. TCR_Tg101_ expanded vigorously in tdLNs of mice with localized tumors derived from parental C1498 cells but were significantly reduced in frequency and number within tdLNs of mice with localized C1498 *K*^*b*−/−^ tumors (Figures 5J–5L). Furthermore, TCR_Tg101_ frequencies and numbers per mg of tumor tissue were significantly higher in mice with parental C1498 versus C1498 *K*^*b*−/−^ tumors (Figures 5M–5O). These results strongly suggested that the Tg101 antigen was directly presented to TCR_Tg101_ by C1498 cells *in vivo*.

### Disparate fates of two leukemia-specific CD8^+^ T cell clones

Finally, the *in vivo* behavior of TCR_Tg101_ and TCR_2C_ was directly compared in leukemia-bearing animals. Because the site and context of initial antigen encounter may be important factors in determining subsequent leukemia-specific CD8^+^ T cell fates, it was of interest to determine the site at which TCR_2C_ and TCR_Tg101_ first encountered antigens *in vivo*. Because our previous work indicated that TCR_2C_ encountered cognate antigen in the spleen very early after leukemia challenge (Kline et al., 2018), TCR_2C_ expansion and CD44 upregulation were assessed 2 days following i.v. C1498.SIY cell challenge (3 days following TCR_2C_ adoptive transfer) in various organs, including the spleen, liver, liver-dLNs (celiac and portal), skin-dLNs, bone marrow, and blood. As predicted, at this early time point, TCR_2C_ had already begun to expand and upregulate CD44 expression almost exclusively in the spleens of leukemia-challenged animals (Figures S6G and S6H). Experiments outlined in Figures 2C–2G suggested that TCR_Tg101_ encountered cognate antigen later than TCR_2C_, likely around 7 to 10 days following C1498 cell challenge. In fact, TCR_Tg101_ proliferation and CD44 upregulation was not detectable in leukemia-challenged mice prior to day 7. However, 7 days following C1498 challenge, prior to proliferation, TCR_Tg101_ began to upregulate CD44 expression to a higher degree in the liver compared to the spleen, bone marrow, LNs, or blood (Figures S6G and S6I). This result suggested that TCR_Tg101_ initially encountered cognate antigen in the liver via direct presentation by C1498 cells (Figure 5).

**Figure 6.**
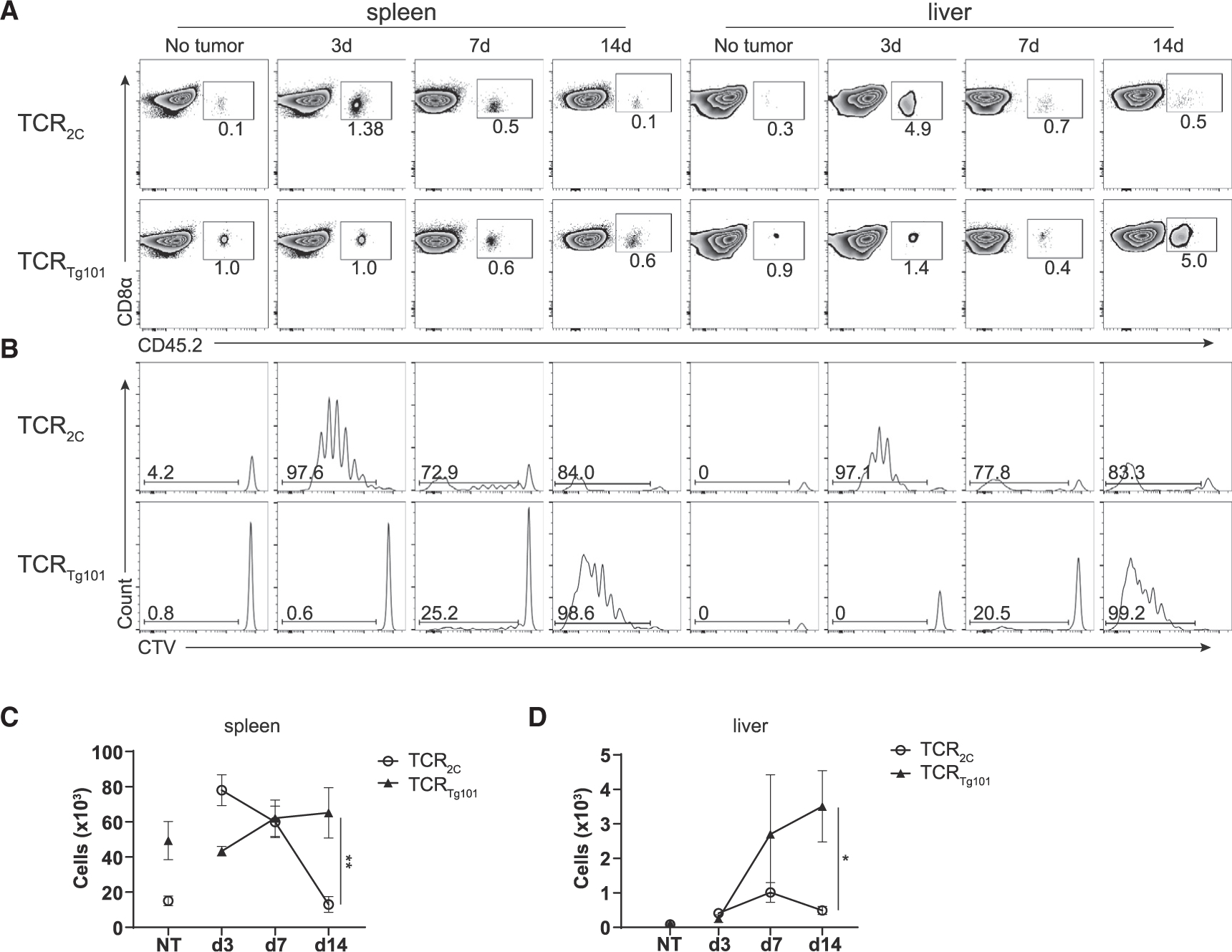
Disparate fates of leukemia-specific CD8^+^ T cell clones CTV-labeled TCR_Tg101_ (CD45.2) or TCR_2C_ (CD45.1.2) (10^6^ each) were adoptively transferred into individual B6.SJL (CD45.1) mice 1 day prior to i.v. challenge with C1498.SIY cells. (A and B) Representative FACS plots depicting the frequencies (A) and CTV dilution profiles (B) of TCR_Tg101_ and TCR_2C_ in spleens or livers of leukemia-bearing mice at the indicated time points. (C and D) Numbers of TCR_Tg101_ and TCR_2C_ in spleens (C) and livers (D) of leukemia-bearing mice at the indicated time points. Data are pooled from 2 independent experiments with 2 to 4 mice/group and presented as mean ± SD. *p < 0.05; **p < 0.01.

As expected, TCR_2C_ expanded rapidly in spleens of mice challenged i.v. with C1498.SIY cells, decreased in number by day 7, and by day 14 were similar in number to TCR_2C_ in leukemia-naive mice (Figures 6A–6C). Similarly, TCR_2C_ expanded minimally by day 7 in livers of leukemia-bearing animals but subsequently decreased to nearly undetectable numbers by day 14 (Figures 6A, 6B, and 6D). These results are consistent with our previous reports showing that TCR_2C_ undergo abortive proliferation and subsequent deletion in mice with leukemia (Kline et al., 2018; Zhang et al., 2013). In stark contrast, TCR_Tg101_ expanded modestly in spleens of mice challenged with parental C1498 cells over time and accumulated significantly in livers of leukemia-bearing mice, particularly when compared with TCR_2C_ (Figures 6A–6D). Thus, although tolerance is effectively established in TCR_2C_ and TCR_Tg101_ in mice with leukemia, the underlying mechanisms are entirely different. Whereas tolerance of TCR_2C_ occurs primarily through deletion requiring antigen cross-presenting cDC1s, TCR_Tg101_ tolerance is mediated by the progressive acquisition of a dysfunctional phenotype requiring direct presentation of the Tg101 antigen by leukemia cells.

## DISCUSSION

The acquisition of a dysfunctional phenotype among antigen-specific CD8^+^ T cells in the solid tumor environment is a well-recognized phenomenon (Gajewski et al., 2006; Thommen and Schumacher, 2018), which in many cases results from chronic TCR stimulation by antigen-expressing cancer cells capable of avoiding immune-mediated elimination (Pauken and Wherry, 2015). This repeated and suboptimal CD8^+^ T cell activation is accompanied by co-inhibitory receptor upregulation, which, along with transcriptional, epigenetic, and metabolic re-programming, leads to a progressive and eventually irreversible exhausted state (Pauken and Wherry, 2015; Philip et al., 2017; Schietinger et al., 2016; Scott et al., 2019; Williams et al., 2017). Because immune checkpoint blockade therapy can restore (at least partially) the function of exhausted CD8^+^ T cells in a subset of patients with cancer (Ribas and Wolchok, 2018; Sharma and Allison, 2015), defining the mechanisms that regulate tumor-specific CD8^+^ T cell tolerance has garnered much attention in the past decade.

In contrast to solid tumors, mechanisms that promote CD8^+^ T cell tolerance to hematologic cancers have not been as well-elucidated. However, we previously reported a unique T cell tolerance mechanism in which antigen cross-presenting cDC1s induced the deletion of high-affinity, leukemia-specific CD8^+^ T cells (TCR_2C_) (Kline et al., 2018; Zhang et al., 2013). Here, we have characterized the behavior of a CD8^+^ T cell clone specific for a naturally expressed leukemia antigen, TCR_Tg101_, which was not deleted but rather accumulated in leukemia-bearing hosts. TCR_Tg101_ expansion, however, was coupled with the acquisition of an exhausted phenotype, characterized by co-inhibitory receptor upregulation, transcriptional reprogramming, and diminished effector function. The markedly disparate fates of TCR_2C_ and TCR_Tg101_ in leukemia-bearing animals might be explained by several factors. First, *in vivo* antigen encounter by TCR_2C_ and TCR_Tg101_ was mediated by different APCs. Leukemia antigen cross-presenting cDC1 were required for antigen encounter by and subsequent deletion of TCR_2C_ (Kline et al., 2018). Conversely, TCR_Tg101_ encountered antigen via direct presentation by C1498 leukemia cells that lack expression of classical T cell co-stimulatory receptors. Second, unique properties associated with the leukemia antigens recognized by TCR_2C_ and TCR_Tg101_ could have conferred the divergent outcomes observed. For example, high-affinity interactions occurring between TCR_2C_ and SIY antigen cross-presenting cDC1s preferentially induced initial proliferation but subsequent deletion of TCR_2C_. On the other hand, presumably lower affinity interactions between TCR_Tg101_ and C1498 leukemia cells, or reduced Tg101 antigen presentation on the leukemia cell surface associated with decreased TCR_Tg101_ avidity, may have promoted their differentiation into an exhausted state (Figure 5A) (Dougan et al., 2013). Finally, the local environments in which these unique CD8^+^ T cell clones encountered their cognate antigens may also have impacted their ensuing fates. TCR_2C_ initially recognized cognate antigen in the spleen (Figures S6G and S6H and Kline et al., 2018), while antigen recognition by TCR_Tg101_ occurred later in the disease course and primarily within the liver, an organ associated with immune-suppressive properties (Kubes and Jenne, 2018). Clearly, further investigation will be required to formally address these important questions independently. Regardless, our results imply that numerous mechanisms underlie antigen-specific CD8^+^ T tolerance in the leukemia-bearing host and suggest that by the point at which a patient is diagnosed with acute leukemia, high-affinity leukemia-specific CD8^+^ T cells may be largely absent, having already been deleted, leaving behind a pool of exhausted, lower-affinity CD8^+^ T cells, the function of which may or may not be restorable with immunotherapeutic intervention.

Another key finding of our work was the inability to restore functional competence of exhausted TCR_Tg101_, which indicated that once fully established, TCR_Tg101_ dysfunction was profound and possibly irreversible. The upregulation of transcription factors in TCR_Tg101_, such as *Tox, Nr4a1, Nr4a2*, and *Nr4a3*, previously shown to mediate epigenetic re-programming of terminally exhausted CD8^+^ T cells in solid cancers (Chen et al., 2019a; Liu et al., 2019; Scott et al., 2019), as well as those known to promote an anergic T cell phenotype, including *Egr2* and *Egr3* (Safford et al., 2005; Zheng et al., 2012), supports the notion that the functionally unresponsive TCR_Tg101_ phenotype at some point becomes fully imprinted as the leukemia progresses in the host. Interestingly, however, even in mice with advanced leukemia, exhausted TCR_Tg101_ largely retained proliferative capability, indicating that the regulation of cell-cycle progression and effector function are uncoupled until very late stages of TCR_Tg101_ dysfunction, which has been previously demonstrated in exhausted CD8^+^ T cells in settings of cancer and chronic viral infection (Schietinger et al., 2016; Wherry, 2011). Furthermore, while upregulation of PD-1 and LAG-3 was observed on the majority of dysfunctional TCR_Tg101_, combined PD-1 and LAG-3 blockade therapy did little to enhance effector cytokine production or control disease progression, which was somewhat surprising given the maintained expression of TCF1 in a significant proportion of TCR_Tg101_ in the leukemia environment (Figures 3G, 3K, and 3L). Regardless, this result indicated that the mechanisms driving the dysfunctional TCR_Tg101_ program were (or at some point became) independent of key T cell checkpoint receptors (Scott et al., 2019; Singer et al., 2016). Finally, despite their functional incompetence, TCR_Tg101_ continued to express mRNAs for effector cytokines (*Ifng*) and cytolytic proteins (*Gzmb, Prf1*) at high levels. Presumably, post-transcriptional mechanisms were negatively regulating translation of these effector molecules, leading to the observed decrease in IFNγ production and cytolytic capacity of dysfunctional TCR_Tg101_ (Salerno et al., 2018). Regardless, this finding supports the notion that the dysfunctional state acquired by TCR_Tg101_ is associated with partially maintained, albeit ineffectual, effector programs. Overall, these results may be important to consider when therapeutic strategies aimed at reversing CD8^+^ T cell dysfunction in the leukemia context are being developed. However, a potential caveat with regard to transplantable murine leukemias is their tendency to progress primarily in the liver compared to bone marrow, as is the case in the human disease. This is a long-standing limitation of such models, and our data should be interpreted with that knowledge in mind.

Surprisingly, classical cross-presentation was dispensable for *in vivo* antigen encounter by TCR_Tg101_. *In vitro* experiments also failed to detect cross-presentation of the Tg101 antigen by professional APCs. On the contrary, direct antigen presentation by leukemia cells was necessary for inducing TCR_Tg101_ expansion and eventual dysfunction *in vivo*. The reasons for the inability of the Tg101 antigen to be displayed through cross-presentation are unknown, but low abundance, or failure of the DC immunoproteasome to efficiently generate the antigenic peptide recognized by TCR_Tg101_, are possible explanations. This question can be directly addressed once the peptide antigen recognized by TCR_Tg101_ has been identified, which is an area of active investigation in our laboratory. Regardless, results outlined in Figures 1 and S1 indicate that the Tg101 antigen is likely leukemia-specific. Once defined, MHC multimers can also be utilized to study the behavior of endogenous CD8^+^ T cells specific for the Tg101 antigen, and the true affinity of TCR_Tg101_ for cognate antigen can be established.

In conclusion, our findings advance the understanding of the mechanisms associated with CD8^+^ T cell tolerance in hematological cancers such as leukemia. We show that the fates of leukemia-specific CD8^+^ T cell clones are highly divergent and are governed, at least in part, by the context in which their cognate antigen is recognized. Regardless of the underlying CD8^+^ tolerance mechanism (deletion versus exhaustion), neither is reversible through immune checkpoint blockade therapy, which may help to explain the disappointing efficacy of anti-PD-1 monotherapy in patients with acute leukemia (Berger et al., 2008). In the context of deletional CD8^+^ T cell tolerance mediated by cDC1s, we have shown that targeting cDC1 activation with toll-like receptor (TLR), CD40, or simulator of interferon genes (STING) agonists can be effective in restoring functional anti-leukemia CD8^+^ T cell responses (Curran et al., 2016; Kline et al., 2018; Zhang et al., 2013). Reinvigorating the function of exhausted, leukemia-specific CD8^+^ T cells may be possible through epigenetic reprogramming (Peng et al., 2015), although this is speculative and will require further experimentation. Finally, we expect that Tg101 mice will be useful to other investigators interested in studying tumor-specific CD8^+^ T cell exhaustion.

### Study limitations

A transplantable murine leukemia model served as the basis for the studies and related conclusions presented above. The extent to which the C1498 leukemia model recapitulates the biology of human leukemia is debatable. Furthermore, although we have thoroughly characterized the behavior of TCR_Tg101_ in leukemia-bearing animals and have identified the cells that present antigen to TCR_Tg101_, without knowing the nature of the Tg101 antigen, we are unable to draw conclusions regarding the natural affinity of this CD8^+^ T cell clone for cognate antigen and the impact on its behavior *in vivo*, nor are we able to identify and investigate the biology of endogenous CD8^+^ T cells reactive to the antigen.

## STAR⋆METHODS

### RESOURCE AVAILABILITY

#### Lead contact

Further information and requests for resources and reagents should be directed to and will be fulfilled by the Lead Contact, Justin Kline, jkline@medicine.bsd.uchicago.edu.

#### Material availability

Tg101 mice generated in the study this study is available from the Lead Contact with a completed Materials Transfer Agreement.

#### Data and code availability

- RNA sequencing data has been deposited at GEO and is publicly available as of the date of publication. Accession number is listed in the key resources table.
- This paper does not report original code.
- Any additional information required to reanalyze the data reported in this paper is available from the lead contact upon request.

### EXPERIMENTAL MODEL AND SUBJECT DETAILS

#### Mice

C57BL/6 mice (H-2^b^; CD45.2) and B6.SJL-*Ptprc*^*a*^/BoyAiTac mice (H-2^b^; CD45.1) were purchased from Taconic Biosciences and breed in our facility. *Rag2*^−/−^ and 2C TCR transgenic mice (Sha et al., 1988) were bred in our facility. CD11cDTR/GFP B6.FVB-Tg (*Itgax*^*DTR-EGFP*^) mice and *Batf3*^−/−^ mice were purchased from Jackson Labs and bred in our facility. Mice were maintained in a specific pathogen-free environment. 6–12 week-old mice, age and sex matched whenever possible, were used for experiments. Animal experimentation was carried out under a protocol approved by an Institutional Animal Use and Care Committee at The University of Chicago.

#### Generation of Tg101 TCR transgenic mice

Tg101 T cell receptor (TCR) transgenic mice were generated in our laboratory in collaboration with the Transgenic/ES cell technology mouse core facility at the University of Chicago. The Tg101 TCR was derived from a C1498 specific CD8^+^ T cell clone (T15) (Boyer et al., 1997). TCR Vα10 (TRAV13) and Vβ1 (TRBV5) genes of the T15 TCR were cloned into pTα and pTβ cassettes (Kouskoff et al., 1995). Tg101 TCR DNA was amplified by PCR using primers for the Vα chain and the Vβ chain (below). The pTα10 and pTβ1 plasmids were linearized using SalI (pTα) and KnpI (pTβ), respectively. Via pronuclear injection, linearized plasmids were separately introduced into fertilized eggs of F1 (C57BL/6J) mice. TCR Vα10 and TCR Vβ1 transgenic founder mice were identified by Vα10 and Vβ1 CDR3 spectratyping. TCR Vα10 and TCR Vβ1 transgenic founder mice were crossed to obtain TCR Vα10/Vβ1 (Tg101) mice. Tg101 founder mice were then crossed onto a *Rag2*^−/−^ background to prevent rearrangement of endogenous TCR loci.

XmaI-intron-Va10 forward:

5’CATCTCCCGGGGCCACACAAGCACCATGAAGAGGCTGCTGTGCTCTCTGC3’

SacII-intron-TRAJ9 reverse:

5’CACCGCGGTAATTTAAATCAAGTTTCTCATTGCACTCACTTGGATCAACCAACAAGCTTGTTCCTG3’

XhoI-intron-TRBV5 forward:

5’AGCCACCTCGAGCCTGATTCCACCATGAGCTGCAGGCTTCTCCTCTATGTTTC3’

SacII-intron-TRBJ1–6 reverse:

5’CTGCAACCGCGGTCAGAAATGGAGCCCCCATACCTGTCACAGTGAGCCGGGTGCCTG3’

#### Cell lines

The C1498 leukemia cell line (H-2^b^) was originally purchased from ATCC. C1498 cells expressing the K^b^-restricted model SIY (SIYRYYGL) peptide antigen were previously generated in our laboratory (Zhang et al., 2009). C1498.B7.1 cells were engineered by retroviral transduction using the pLEGFP-B7.1 plasmid. B16.F10 and EL4 cell lines were provided by Dr. Thomas Gajewski. Cell lines were routinely tested for mycoplasma contamination using a VenorTM GEM mycoplasma detection kit (Sigma). The *H2-K*^*b*^-deficient (*K*^*b*−/−^) C1498 cell line was generated as previously described (Kline et al., 2018). The *H2-D*^*b*^-deficient (*D*^*b*−/−^) C1498 cell line was generated via CRISPR/Cas9 targeting using the following guide sequences: forward - CACCGACCCGCGCGGGTCTGAGTCG; reverse - AAACCGACTCAGACCCGCGCGGGTC.

All the cell lines described in this manuscript are of a H2^b^ haplotype. C1498 derivative cell lines and B16.F10 cells were cultured in DMEM, and EL4 cells were cultured in RPMI-1640 (Invitrogen) both supplemented with 10% FBS, 2-mercaptoethanol, essential amino acids, penicillin and streptomycin at 37°C.

### METHOD DETAILS

#### Flow cytometry

Spleens, lymph nodes, livers, peripheral blood, and bone marrow were harvested from mice, mechanically dissociated with a 3 mL syringe plunger, and pushed through 70 um mesh filter to generate single cell suspensions (spleens, livers, lymph nodes). Liver mononuclear cells were enriched over a Ficoll gradient (BD Biosciences). Following red blood cell lysis and F_c_ receptor blockade using anti-CD16/32 antibodies, cells were stained with the following directly conjugated antibodies (clones): CD45.2 (104), CD45.1 (A20), CD3ε (145–2C11), TCRβ (H57–597), CD8α (53–6.7), CD4 (RM4.5), Thy1.2 (30-H12), CD11b (M1/70), H2-D^b^ (KH95), H2-K^b^ (AF6–88.5), I-A^b^/I-E^b^ (M5/114.15.2), PD-1 (RMP1–30), TIM3 (RMT3–23), LAG-3 (C9B7W), TIGIT (GIGD7), TNFα (Mp6-XT22), IFNγ (XMG1.2), Granzyme B (QA16A02), B220 (RA3–6B2), CD44 (IM7), CD62L (Mel-14), CD69 (H1.2F3), CD11c (HL3), Eomes (Dan11mag), T-bet (4B10), Egr2 (erongr2) and TOX (REA473). Fixable viability dyes (Invitrogen) were used to exclude dead cells. Flow cytometry was performed on LSRII or LSRFortessa cytometers (BD Biosciences). Analysis was performed using FlowJo software (Treestar). Fluorescence-activated cell sorting (FACS) was performed using a FACSAria (BD Biosciences). Intracellular antibody staining was performed using a Foxp3 staining kit (eBioscience).

#### T cell labeling with CellTrace Violet

TCR_Tg101_ were enriched from spleens of Tg101 mice using a mouse CD8 microbead kit (Miltenyi). Isolated TCR_Tg101_ were washed once with PBS and labeled with CellTrace Violet (CTV) (Invitrogen) for 20 minutes at 37°C and quenched with 10% FBS in RPMI-10. Prior to adoptive transfer, CTV-labeled TCR_Tg101_ were washed twice more with PBS.

#### *In vitro* TCR_Tg101_ proliferation assay

10^5^ CTV-labeled TCR_Tg101_ were plated in 96 well, flat-bottom tissue culture plates with (or without) 2×10^4^ γ-irradiated tumor cells (12,000 rads), or with freshly-harvested spleen cells from C57BL/6 mice, and cultured at 37°C for 3 days. Cells were then harvested, and TCR_Tg101_ proliferation, measured by CTV dilution, was analyzed by flow cytometry.

#### *In vivo* TCR_Tg101_ proliferation assay

1–2×10^6^ CTV-labeled, congenically-marked TCR_Tg101_ or TCR_2C_ were adoptively transferred i.v. through the lateral tail vein into B6.SJL mice (CD45.1) or into C57BL/6 mice (CD45.2). One day later, 10^6^ C1498 cells (or derivative C1498 cell lines) were inoculated i.v. At subsequent time points indicated, TCR_Tg101_ number, proliferation, co-inhibitor receptor expression, and effector function was analyzed in spleens and livers by flow cytometry. TCR_2C_ numbers were assessed similarly.

#### *In vitro* antigen cross-presentation assay

Bone marrow cells isolated from femurs of B6.SJL mice (CD45.1) were cultured with 40 ng/ml granulocyte-macrophage colony-stimulating factor (GM-CSF; Biolegend). One-half of the medium was removed on day 2 and replaced with fresh GM-CSF-supplemented medium warmed to 37°C. On day 3, the culture medium was discarded and again replaced with fresh, warmed, GM-CSF-supplemented medium (20 ng/ml). This process was repeated until day 8. At this point, non- and loosely adherent cells (BMDCs) were harvested by gentle washing with PBS and plated at a concentration of 1×10^5^ cells/well in round-bottom 96-well plates. BMDCs were then pretreated with 20 ng/ml LPS for 2 hours and pulsed with C1498 or C1498.SIY cell lysates (3 tumor cell lysates to 1 BMDC). Next, CTV-labeled, congenically-marked TCR_2C_ or TCR_Tg101_ were added and cultured at 37°C for 3 days. Cells in each well were then harvested, and CTV dilution of TCR_2C_ or TCR_Tg101_ was analyzed by flow cytometry as a read out for *in vitro* antigen cross-presentation.

#### *Ex vivo* antigen cross-presentation assay

8×10^6^ C1498.SIY cells were injected i.v. into B6.SJL mice. Three hours later, spleens were harvested and injected with 1 mg/ml collagenase IV (Sigma),20 mg/ml DNase I (Roche) in RPMI with 2% FBS and were incubated at 37°C for 15–20 minutes. Spleens were then dissociated with a 3 mL plunger and passed through a 70 um mesh filter to generate single cell suspensions. CD3^+^ and CD19^+^ cells were depleted by positive selection. Remaining cells were stained with fluorescently-labeled antibodies directed against CD3ε (145–2C11), CD19(eBio1D3), CD11c (HL3), CD8α (53–6.7), and CD11b (M1/70). After excluding CD3^+^ and CD19^+^ cells, CD8α^+^ DCs (cDC1) CD11b^+^ DCs (cDC2), and CD11b^+^CD11c^lo/−^ (monocytes/macrophages) cells were separately isolated by FACS. Sorted APC populations were cultured 1:1 with purified, congenically-marked, CTV-labeled TCR_2C_ or TCR_Tg101_ for 65–72 hours in 96 well, round bottom tissue culture plates. Subsequently, CTV dilution of TCR_2C_ or TCR_Tg101_ was assessed by flow cytometry as a read out for *ex vivo* antigen cross-presentation.

#### *Ex vivo* TCR_Tg101_ restimulation and intracellular cytokine staining

Mononuclear cells isolated from livers of mice at the indicated time points following C1498 cell inoculation were restimulated with plate-bound anti-CD3 antibody (clone: 145–2C11; 1 μg/ml) plus soluble anti-CD28 antibody (clone PV-1, 1 ug/ml), or with PMA plus ionomycin, for 1 hour, followed by 4 hours in the presence of 1 mg/ml GolgiPlug (BD Biosciences). Intracellular staining of effective cytokines was performed using FoxP3 intracellular staining kit (eBiosciences). In some experiments, irradiated (12,000 rads) C1498.B7.1 cells were utilized to restimulate TCR_Tg101_ *ex vivo* overnight prior to intracellular cytokine staining and flow cytometric analysis.

#### *Ex vivo* TCR_Tg101_ killing assay

CTV^hi^ or CTV^lo^ TCR_Tg101_ were separately FACS-purified from livers of mice 17–18 days after i.v. C1498 cell inoculation and 1×10^4^ cells were cultured *in vitro* with equal numbers of C1498 cells and EL4 cells (1×10^4^ each) labeled with two different concentrations of CTV, along with IL-2 (50 U/ml) and IL-12 (20 ng/ml) in 384 well round bottom tissue culture plates. In negative control wells, C1498 cells and EL4 cells were plated in the absence of TCR_Tg101_. Approximately 20 hours later, proportions of surviving C1498 and EL4 cells in each well were analyzed by flow cytometry. Naive TCR_Tg101_ isolated from spleens of Tg101 mice were activated *in vitro* for 72 hours with anti-CD3 and anti-CD28 antibodies or irradiated C1498.B7.1 cells prior to co-culture with C1498 cells and EL4 cells to serve as positive controls for C1498 cell lysis. Naive, unstimulated TCR_Tg101_ isolated from spleens of Tg101 mice were also co-cultured with C1498 cells and EL4 cells to serve as a negative control for C1498 cell lysis. Specific lysis was calculated as follows (Noto et al., 2013):

$$\%\;specific\;lysis=100-\frac{C1498\;:\;EL4\;\left(with\;TCRTg101\right)}{C1498\;:\;EL4\;\left(without\;TCRTg101\right)}\times 100$$

#### Generation of bone marrow chimeric (BMC) mice

C57BL/6 mice received total body irradiation (900 rads) and were reconstituted 16 hours later with 2.5×10^6^ bone marrow cells isolated from CD11c-DTR mice. Eight weeks later, these bone marrow chimeric mice received TCR_Tg101_ adoptive transfer, followed 1 day later by an i.v. C1498 cell challenge. To deplete CD11c^+^ cells from leukemia-bearing CD11c-DTR bone marrow chimeric mice, diphtheria toxin (DT) (500 ng) was administered i.p. 2 days prior to C1498 cell inoculation and continued every 48 hours for a total of 7 doses.

#### Survival experiments

10^6^ C1498 cells were inoculated i.v. into C57BL/6 mice three days before adoptive transfer of 4×10^6^ naive or anti-CD3 and anti-CD28 activated TCR_Tg101_ or TCR_2C_. Control groups of C57BL/6 mice received an i.v. C1498 cell challenge but did not receive TCR_Tg101_ or TCR_2C_ adoptive cell transfer. Survival was monitored. In experiments involving immune checkpoint blockade therapy, mice transferred with TCR_Tg101_ and challenged with C1498 cells were subsequently administered anti-PD-1 (RMP1–14) and anti-LAG-3 (C9B7W) antibodies (BioXcell) intra-peritoneal, 200 μg each, beginning on day 6, and continued every other day for 2 weeks. Control mice received isotype control antibodies at the same dose and schedule. Survival was monitored.

#### RNA sequencing

TCR_Tg101_ in livers and spleens of leukemia-bearing mice were isolated at various time points following adoptive transfer (day 0, day 6–7, day 13–14, day 16–18) by FACS and were re-suspended in Trizol (Life Technologies). TCR_Tg101_ RNA was isolated via chloroform extraction. Low input RNA sequencing was performed in the University of Chicago Genomic Core Facility on the Illumina HiSeq 2500 platform in two batches. Reads were mapped onto the University of California Santa Cruz mouse genome using kallisto (Bray et al., 2016). Genes with fewer than 10 reads in at least 6 samples were filtered out, resulting in a dataset of 11,164 genes. Differential gene expression analysis was performed on raw aligned read counts using DESeq2 (Love et al., 2014), with batch effects accounted for in the design formula. Genes were considered to be differentially expressed if they had an adjusted p value < 0.05 using a Benjamini-Hochberg test (FDR). Counts per gene were regular log (rlog)-transformed, and batch effects were removed using the removeBatch-Effect function from *limma* (Ritchie et al., 2015) for principal component analysis (PCA), sample clustering based on Euclidean distance, and heatmaps depicting z-scores of gene expression. rlog-transformed, batch-corrected counts were also used for weighted gene correlation network analysis (WGCNA) (Langfelder and Horvath, 2008) while additionally filtering out the 50% of genes with the lowest variance to reduce noise, resulting in a set of 5,582 genes. Adjacency was determined using a signed analysis and a soft thresholding power of 14, which was determined by scale-free fit index (Zhang and Horvath, 2005). 12 clusters of genes were initially identified, 2 of which contained a combined 4,269 genes (76.5% of the dataset), and roughly corresponded to genes whose expression increased or decreased over the experimental time course. Of the other 10 clusters, 2 were identified as containing genes which were transiently upregulated or downregulated. These two clusters contained 448 genes, combined. The remaining 865 genes were assigned to eight different clusters, which appeared to be the result of high variance within sample groups, either due to low overall gene expression or low outlier values. No conclusions were drawn regarding these clusters due to uncertainty in the data.

### QUANTIFICATION AND STATISTICAL ANALYSIS

Grouped data were analyzed via two-way ANOVA with Bonferroni post-tests. Survival differences were analyzed with the Log-rank test. Statistics were performed using GraphPrism software. Data are presented as mean ± SD unless otherwise indicated. A p value of < 0.05 was considered statistically significant. Additional description of statistical methods for individual experiments can be found in the figure legends.

## Supplementary Material

1

2

## Figures and Tables

**KEY RESOURCES TABLE:** 

REAGENT or RESOURCE	SOURCE	IDENTIFIER

Antibodies		

CD45.2	Biolegend	Clone: 104; Cat# 109820; 109806; 109814
CD45.1	Biolegend	Clone: A20; Cat#110722; 110706; 110708; 110714
CD3e	Biolegend, BioxCell	Clone: CD3e (145–2C11); Cat#100204; BE0001–1
TCRβ	Biolegend	Clone: H57–597;Cat#109208
CD8α	Biolegend; eBioscience	Clone: 53–6.7; Cat# 100744; 100734; Cat #45–0081-82
CD4	Biolegend	Clone:RM4.5; Cat#100528
Thy1.2	Biolegend	Clone: 30-H12; 105326
CD11b	Biolegend	Clone:M1/70; Cat#101263
H2-D^b^	Biolegend	Clone:KH95; Cat# 111507
H2-K^b^	Biolegend	Clone: AF6–88.5; Cat#116518
I-A^b^/I-E^b^	eBioscience	Clone: (M5/114.15.2); Cat#17–5958-82
PD-1	eBioscience	Clone: RMP1–30; Cat#25–5982-82
TIM3	Biolegend	Clone: RMT3–23; Cat#119704
LAG-3	Biolegend; BioXcell	Clone: C9B7W; Cat#125210; BE0174
TIGIT	eBioscience	Clone:GIGD7;Cat# 12–9501-82
TNFα	eBioscience	Clone:Mp6-XT22; Cat#12–7321-82
IFNγ	BD	Clone: XMG1.2; Cat#554413
Granzyme B	Biolegend	Clone: QA16A02; Cat#372211
B220	Biolegend	Clone:RA3–6B2; Cat#103206
CD44	Biolegend	Clone: IM7; Cat#103030
CD62L	Biolegend	Clone: Mel-14;
CD69	Biolegend	Clone: H1.2F3;
CD11c	Biolegend	Clone: HL3;
Eomes	Biolegend	Clone: Dan11mag;
T-bet	Biolegend	Clone: 4B10;
Egr2	Biolegend	Clone: erongr2;
TOX	Miltenyi	Clone: REA473; Cat#130–118-474
CD28	BioXCell	Clone: PV-1; Cat#BE0015–5
CD16/32	Bio X Cell	2.4g2;Cat# BE0307

Chemicals, peptides, and recombinant proteins		

PMA	Sigma	Cat# P1585
ionomycin	Sigma	Cat# I0634
CellTrace Violet Cell Proliferation Kit, for flow cytometry	Invitrogen	Cat# C34557
APC BrDU Kit	BD	Cat# 552598
Foxp3 staining kit	eBioscience	Cat#00–5523–00
LIVE/DEAD Fixable Near-IR Dead Cell Stain Kit	Invitrogen	Cat#L10119
1X DPBS GIBCO 14190–250	GIBCO 14190–250	Cat#14190–250
RPMI 1640	GIBCO	Cat#11875–119
DMEM	GIBCO	Cat#11965–118
Fetal Bovine Serum	Gemini	Cat#100–106
Penicillin/Streptimycin	GIBCO	Cat#15140–122
0.05% Trypsin EDTA		Cat#25300062
Dnase I	Roche	Cat#10104159001
Collgenase IV	Sigma	Cat#C5138
IL-2	Biolegend	Cat#575402
IL-7	Peprotech	Cat#217–17
IL-15	Biolegend	Cat#566301
GM-CSF	Biolegend	Cat#576304
CD8a Microbeads (mouse)	Miltenyi	Cat#130–117–044
Venor GeM Mycoplasma Detection Kit, PCR-based	Sigma	Cat#MP0025–1KT

Experimental models: Cell lines		

C1498	ATCC	Cat#TIB-49
C1498.SIY	This lab	Zhang et al., 2009
C1498.B7.1	This paper	N/A
B16.F10	A gift from Thomas Gajewski, University of Chicago	N/A
EL4	A gift from Thomas Gajewski, University of Chicago	N/A
C1498.*H2-K^b−/−^*	This lab	Kline et al., 2018
C1498.*H2-D^b−/−^*	This paper	N/A

Experimental models: Organisms/strains		

C57BL/6	Taconic	Cat# B6
B6.SJL-*Ptprc*^a^/BoyAiTac	Taconic	Cat# 4007
*Rag2^−/−^*	Taconic	Cat# RAGN12
2C TCR transgenic mice	Thomas Gajewski lab	Sha et al., 1988
CD11cDTR/GFP B6.FVB-Tg (*Itgax^DTR-EGFP^*)	Jackson Laboratory	Cat# 004509
*Batf3^−/−^*	Jackson Laboratory	Cat# 013755

Oligonucleotides		

Primer XmaI-intron-Va10 forward	IDT	5′CATCTCCCGGGGCCACACAAG CACCATGAAGAGGCTGCTGTGCTCTCTGC3′
Primer SacII-intron-TRAJ9 reverse	IDT	5′CACCGCGGTAATTTAAATCAAGT TTCTCATTGCACTCACTTGGA TCAACCAACAAGCTTGTTCCTG3′
Primer XhoI-intron-TRBV5 forward	IDT	5′AGCCACCTCGAGCCTGATTCCACCATG AGCTGCAGGCTTCTCCTCTATGTTTC3′
Primer SacII-intron-TRBJ1–6 reverse	IDT	5′CTGCAACCGCGGTCAGAAATGGAGCCCC CATACCTGTCACAGTGAGCCGGGTGCCTG3′

Recombinant DNA		

pTα and pTβ cassettes	Diane Mathis lab	Kouskoff et al., 1995
pLEGFP-N1	Clontech	6059–1

Software and algorithms		

Flowjo_V10	Treestar	Version 10; https://www.flowjo.com/
Prism	GraphPad	Version 8; https://www.graphpad.com/scientific-software/prism/
Adobe Illustrator	Adobe	Version 25.3.1, https://www.adobe.com/

Deposited data		

RNA-Seq	This paper	GEO # GSE186268

## Highlights

- A native leukemia antigen-specific TCR transgenic mouse strain (Tg101) is generated
- Leukemia-specific T cells (TCR_2C_) are deleted by CD8α^+^ DCs
- TCR_Tg101_ became dysfunctional via direct interactions with leukemia cells
- Distinct modes of leukemia antigen display confer unique CD8^+^ T cell tolerant states
