# Supplementary material for: Divergent fates of antigen-specific CD8+ T cell clones in mice with acute leukemia
Source: Cell Rep. Author manuscript; Available in PMC 2021 Dec 9. (PMC8656370; doi:10.1016/j.celrep.2021.109991)
Supplement: 1 [file NIHMS1756258-supplement-1.pdf]

Cell Reports, Volume *37*

## Supplemental information

# Divergent fates of antigen-specific CD8$^{+}$ T cell clones in mice with acute leukemia

**Xiufen Chen, Brendan W. MacNabb, Blake Flood, Bruce R. Blazar, and Justin Kline**

Supplementary Figure 1

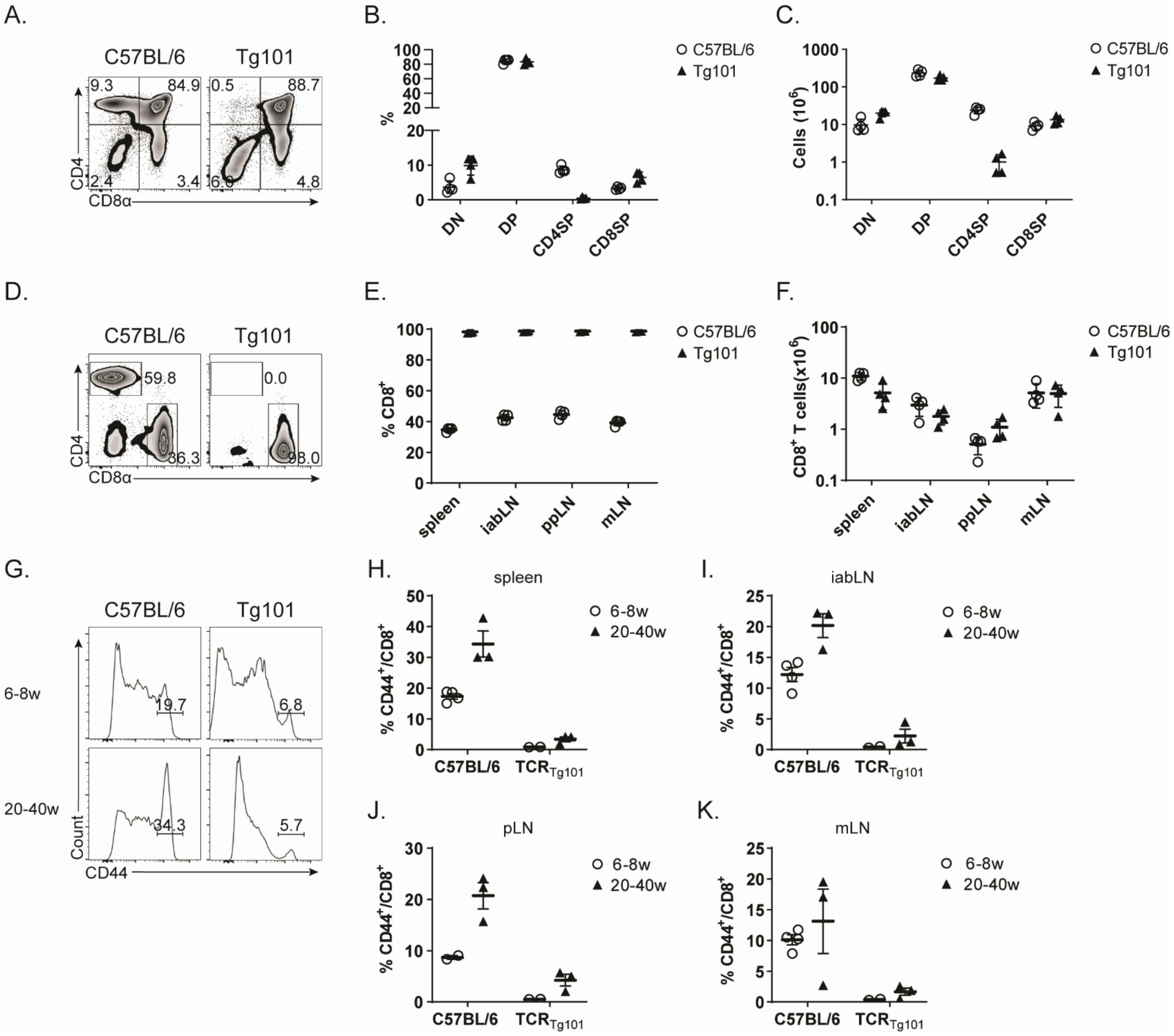


**Supplementary Figure 1. T cell phenotyping of Tg101 TCR transgenic mice. Related to Figure 1.** (**A-C**) Thymocyte development in *Rag2*$^{-/-}$ Tg101 mice**.** CD4 and CD8 expression on live thymocytes from 6-8 week-old C57BL/6 and *Rag2*$^{-/-}$ Tg101 mice were analyzed by flow cytometry. Representative FACS plots are shown in (**A**). Quantified data are shown in (**B and C**) as mean ± SD. DN – double negative; DP – double positive; SP - single positive. (**D-F**) Frequencies and absolute numbers of CD4$^{+}$ and CD8$^{+}$ T cells in SLO of C57BL/6 and *Rag2*$^{-/-}$ Tg101mice (gated on CD3$^{+}$ cells). Representative FACS plots are shown in (**D**) and quantified results are shown in

(**E and F**). Sp - spleen; iabLN - pooled inguinal, axillary and brachial lymph lodes; pLN – periaortic lymph node; mLN - mesenteric lymph lode. (**G-K**) Analysis of CD44 expression on $CD8^+$ T cells from C57BL/6 and *Rag2*$^{-/-}$ Tg101 mice. Representative FACS plots depicting the frequency of CD44-expressing splenic $CD8^+$ T cells from younger (6-8 weeks) and older (20-40 weeks) mice are shown in (**G**). Summary plots of different SLO are shown in (**H-K**) as mean ± SD. Data are representative of at least 2 experiments with 2-4 mice/group.

Supplementary Figure 2

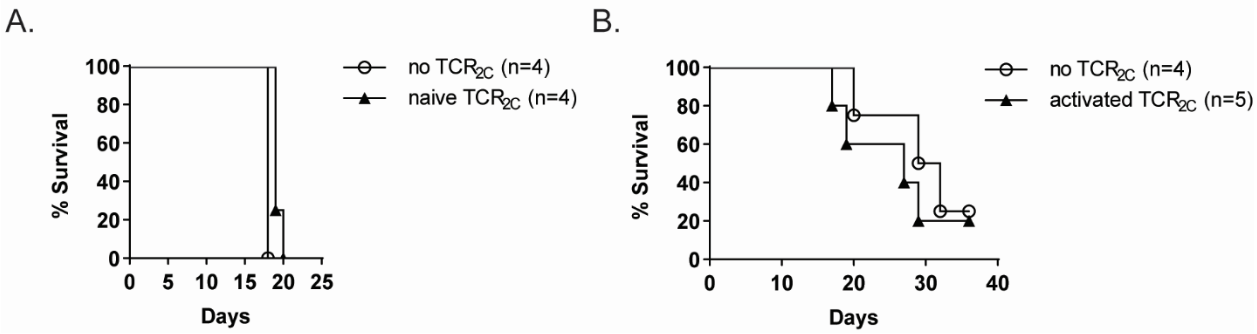


**Supplementary Figure 2. Adoptive transfer of naïve or activated $TCR_{2C}$ does not impact survival of mice with leukemia. Related to Figure 2.** (**A and B**) Survival of C57BL/6 mice challenged i.v. with C1498.SIY cells ($10^6$) and transferred or not with naive (**A**) or *in vitro* activated (**B**) $TCR_{2C}$ ($4x10^6$) 3-5 days later.

Supplementary Figure 3

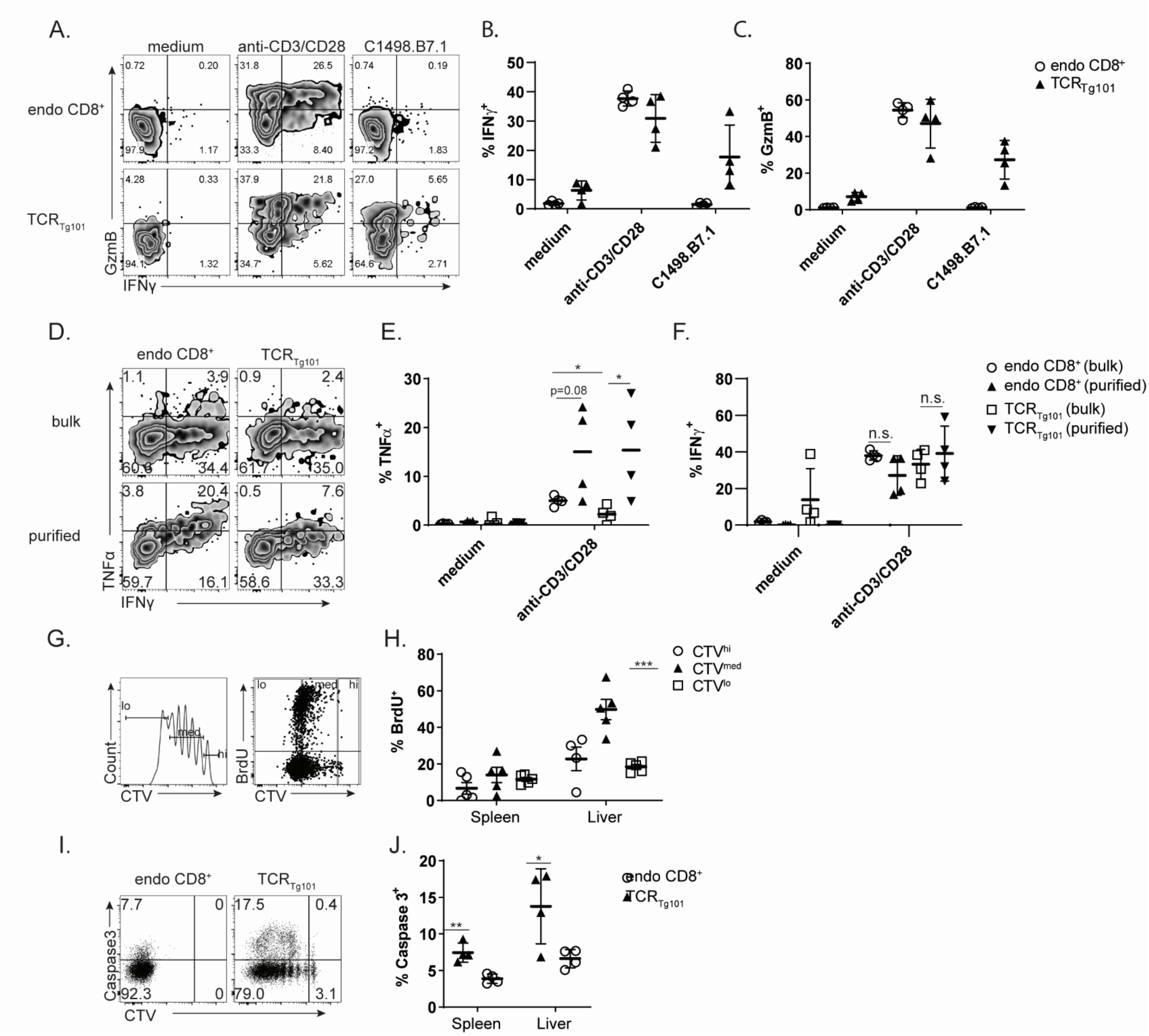


**Supplementary Figure 3. TCR$_{Tg101}$ effector cytokine, proliferation and apoptosis profiles. Related to Figure 2.** (**A-C**) Mononuclear cells isolated from livers of mice 18 days after C1498 cell inoculation (19 days following TCR$_{Tg101}$ (CD45.1.2) adoptive transfer) were restimulated with anti-CD3 and anti-CD28 antibodies or irradiated C1498.B7.1 cells with IL-2 (50 U/ml), IL-7 (5 ng/ml), and IL-12 (20 ng/ml) overnight. Cytokine production was analyzed by flow cytometry. Gating was performed on TCRβ$^+$CD8$^+$CD45.1 cells (endogenous CD8$^+$ T cells) or TCRβ$^+$CD8$^+$CD45.1.2 cells (TCR$_{Tg101}$). (**A**) Representative FACS plots showing IFNγ and Granzyme B (GzmB)

production by endogenous $CD8^+$ T cells or $TCR_{Tg101}$. Quantitative data are shown in (**B and C**) as mean ± SD. (**D-F**) $CD8^+$ T cells (endogenous and $TCR^{Tg101}$) were purified or not from Ficoll-enriched mononuclear cells from livers of mice 18 days after C1498 cell inoculation. Bulk mononuclear cells or purified $CD8^+$ T cells were restimulated with anti-CD3 and anti-CD28 antibodies plus IL-2 and IL-12 overnight, and cytokine production was analyzed by flow cytometry. Gating was performed on $TCR\beta^+CD8^+$CD45.1 cells (endogenous $CD8^+$ T cells) or $TCR\beta^+CD8^+$CD45.1.2 cells ($TCR_{Tg101}$). (**D**) Representative FACS plots showing TNFα and IFNγ production by endogenous $CD8^+$ T cells or $TCR_{Tg101}$ from bulk or purified $CD8^+$ T cell populations. Quantitative data are shown in (**E and F**) as mean ± SD. * $p < 0.05$; n.s. – not significant (**G and H**) BrdU incorporation by CTV-labeled $TCR_{Tg101}$ in livers of leukemia-bearing mice 17 days after C1498 cell challenge. (**G**) Representative FACS plots showing $CTV^{hi}$, $CTV^{med}$, and $CTV^{low}$ $TCR_{Tg101}$ populations (left) and BrdU incorporation by CTV dilution of $TCR_{Tg101}$ (right). Gating was performed on $TCR\beta^+CD8^+$CD45.2 cells. (**H**) Quantified data from (**G**) as mean ± SD. (**I** and **J**) Activated caspase 3 in endogenous $CD8^+$ T cells or $TCR_{Tg101}$ from spleens or livers of leukemia-bearing mice 17 days following C1498 cell challenge. (**I**) Representative FACS plots showing activated caspase 3 expression in endogenous $CD8^+$ T cells and $TCR_{Tg101}$ in livers of leukemia-bearing animals. (**J**) Quantified data shown as mean ± SD. Data are pooled from 2 experiments with 2-3 mice/group. * $p < 0.05$; ** $p < 0.01$. *** $p < 0.001$. n.s. – not significant.

Supplementary Figure 4

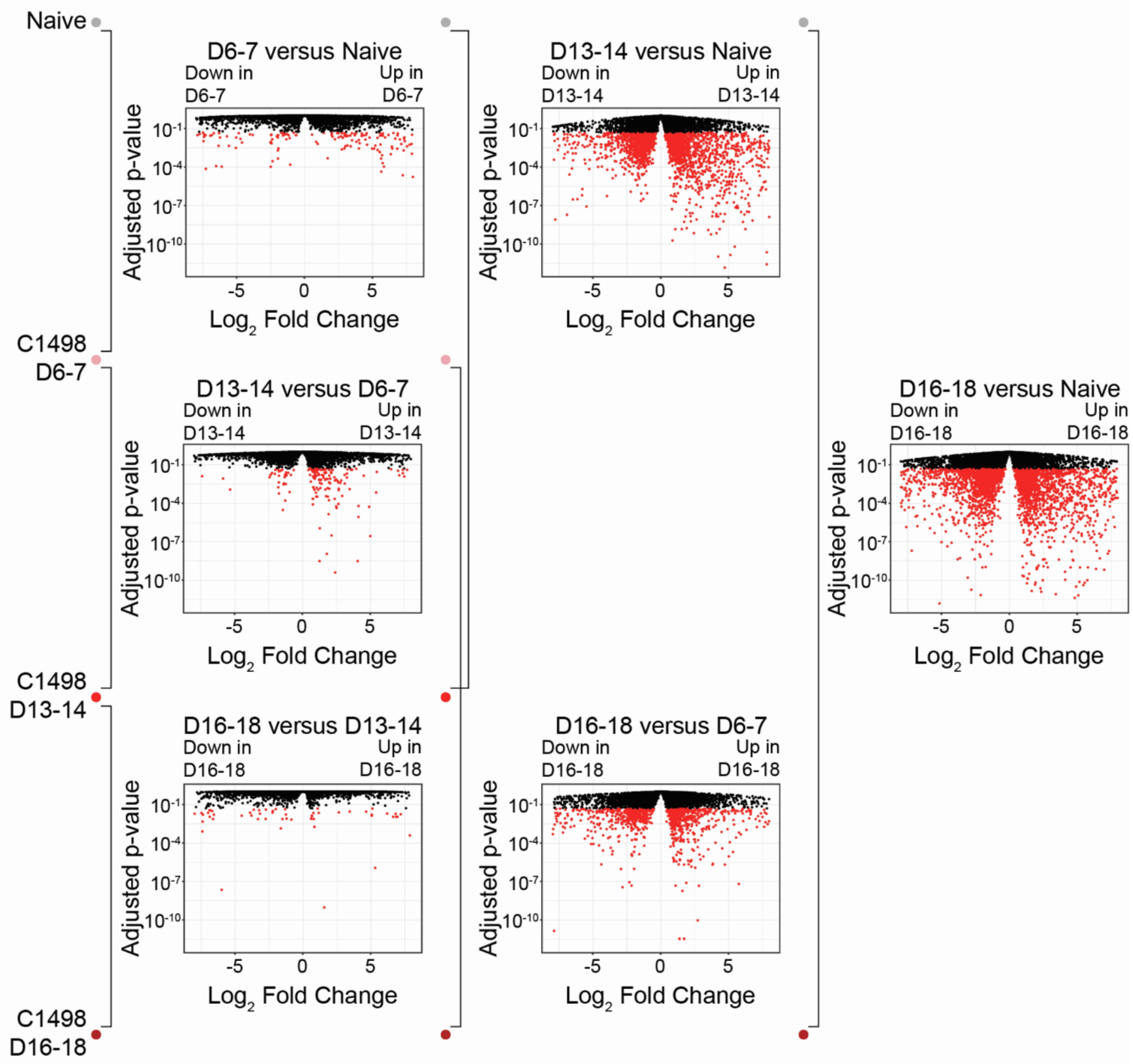


**Supplementary Figure 4. Volcano plots showing differentially expressed genes in $TCR_{Tg101}$ at specific time points. Related to Figure 3.** Pairwise comparisons of RNA-seq profiles of $TCR_{Tg101}$ from leukemia-bearing mice at early (day 6-7, pink icon), mid (day 13-14, red icon), and late (day 16-18, maroon icon) time points, as well as naïve mice (grey icon). Genes with an adjusted p-value <0.05 (Benjamini-Hochberg) were considered to be differentially expressed and are depicted in red.

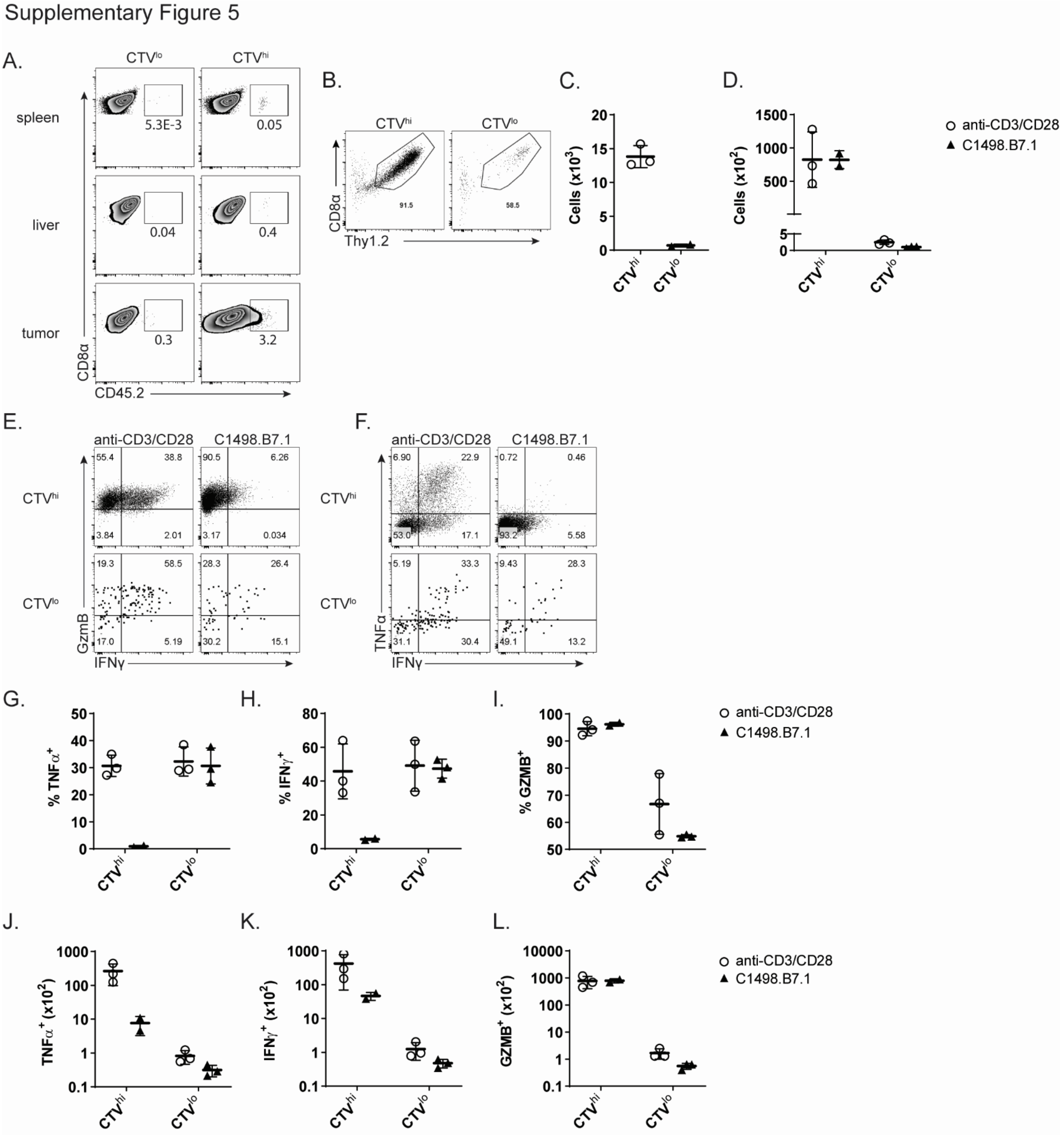


**Supplementary Figure 5. The dysfunctional $TCR_{Tg101}$ phenotype is not readily reversible. Related to Figure 4. (A)** Identification of naïve ($CTV^{hi}$) or exhausted ($CTV^{lo}$) $TCR_{Tg101}$ in secondary hosts. Experimental design outlined in Figure 4A. Representative FACS plots showing frequencies of naïve ($CTV^{hi}$) and exhausted ($CTV^{lo}$) $TCR_{Tg101}$ (CD45.1.2) in the indicated tissues of secondary B6.SJL mice (CD45.1) with s.c. C1498 tumors. (**B and**

**C**) CTV$^{hi}$ (naïve) and CTV$^{lo}$ (exhausted) $TCR_{Tg101}$ were FACS-purified from livers of leukemia-bearing mice17-18 days after i.v C1498 cell challenge, and ($1x10^4$) were cultured with of IL-2 (50 U/ml), IL-7 (5 ng/ml), IL-12 (20 ng/ml) and IL-15 (5 ng/ml) for 5 days, at which point, surviving $TCR_{Tg101}$ were enumerated. (**B**) Representative FACS plots showing live $TCR_{Tg101}$ from CTV$^{hi}$ and CTV$^{lo}$ populations. Quantified data are shown in (**C**). (**D-L**) CTV$^{hi}$ and CTV$^{lo}$ $TCR_{Tg101}$ were FACS-purified from livers of leukemia-bearing mice 17-18 days after i.v C1498 cell challenge, and 2-$3x10^4$ CTV$^{hi}$ or CTV$^{lo}$ $TCR_{Tg101}$ were restimulated with anti-CD3 and anti-CD28 antibodies or irradiated C1498.B7.1 cells in the presence of IL-2 (50 U/ml) and IL-12 (20 ng/ml) for 5 days. Viability and cytokine production by CTV$^{hi}$ and CTV$^{lo}$ $TCR_{Tg101}$ was analyzed by flow cytometry. (**D**) Quantification of live $TCR_{Tg101}$ following 5 days of *in vitro* culture. (**E and F**) Representative FACS plots showing effector cytokine production and GzmB expression among *in vitro* cultured CTV$^{hi}$ and CTV$^{lo}$ $TCR_{Tg101}$ following restimulation with anti-CD3 and anti-CD28 antibodies or with irradiated C1498.B7.1 cells. (**G-I**) Quantified data showing frequencies of IFNγ-, TNFα-, or dual cytokine-producing, viable CTV$^{hi}$ and CTV$^{lo}$ $TCR_{Tg101}$ after restimulation. (**J-L**) Quantified data showing absolute numbers of IFNγ-, TNFα-, or dual cytokine-producing, viable CTV$^{hi}$ and CTV$^{lo}$ $TCR_{Tg101}$ after restimulation. Data are representative of 2 experiments with 2-3 mice/group and shown as mean ± SD.

Supplementary Figure 6

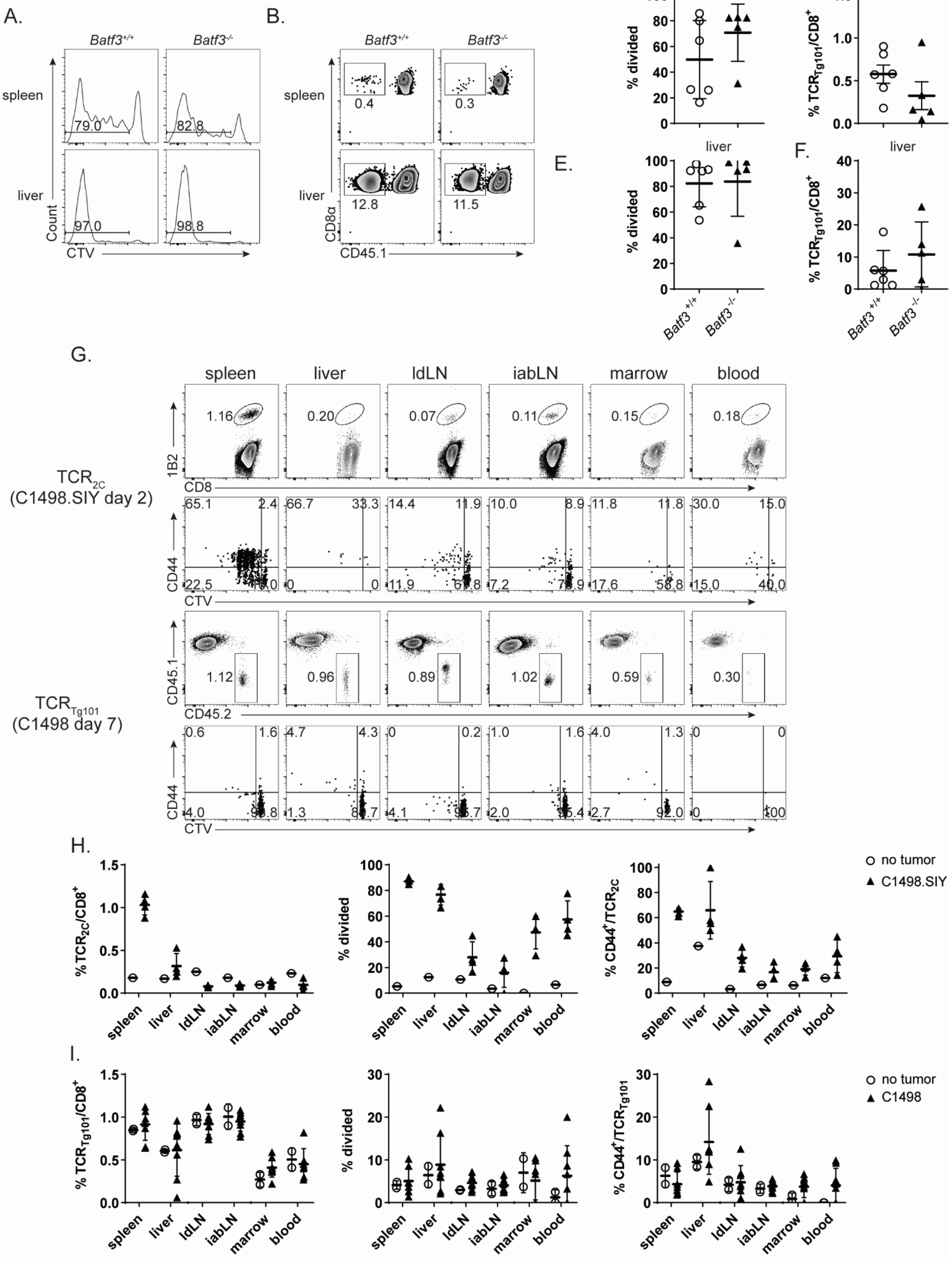

**Supplementary Figure 6. $TCR_{Tg101}$ initially encounter antigen in the liver in a cDC1-independent manner. Related to Figures 5 and 6.** (**A-F**) CTV-labeled $TCR_{Tg101}$ (CD45.2) were adoptively transferred into *Batf3*$^{+/+}$ (CD45.1) or *Batf3*$^{-/-}$ (CD45.1.2) mice 1 day prior to i.v. challenge with C1498 cells. Fifteen days later, $TCR_{Tg101}$ proliferation and frequencies in spleens and livers of *Batf3*$^{+/+}$ and *Batf3*$^{-/-}$ mice were analyzed by flow cytometry. Representative FACS plots showing CTV dilution (**A**) or frequency (**B**) of $TCR_{Tg101}$ in spleens and livers of leukemia-bearing *Batf3*$^{+/+}$ and *Batf3*$^{-/-}$ mice. (**C-F**) Quantitative data showing percent divided $TCR_{Tg101}$ (**C and E**) and $TCR_{Tg101}$ frequencies (**D and F**) in spleens (**C and D**) and livers (**E and F**) of leukemia-bearing *Batf3*$^{+/+}$ versus *Batf3*$^{-/-}$ mice. Data in **C-E** are pooled from 2 independent experiments with 2-3 mice/group and shown as mean ± SD. (**G-I**) $1x10^6$ CTV-labeled $TCR_{2C}$ or $TCR_{Tg101}$ were adoptively transferred into groups of congenic C57BL/6 mice that received an i.v. challenge with C1498.SIY cells ($TCR_{2C}$) or C1498 cells ($TCR_{Tg101}$) the following day, or that received no subsequent leukemia cell challenge. Two ($TCR_{2C}$) or seven ($TCR_{Tg101}$) days later, spleens, livers, bone marrow, pooled skin-draining lymph nodes, pooled liver-draining lymph nodes (portal and celiac), and peripheral blood were harvested. Frequencies, proliferation profiles, and CD44 expression among $TCR_{2C}$ or $TCR_{Tg101}$ at the indicated sites were analyzed by flow cytometry. (**G**) Representative FACS plots showing $TCR_{2C}$ (top) or $TCR_{Tg101}$ (bottom) frequency, proliferation (CTV dilution) and CD44 expression at the sites indicated. (**H and I**) Quantitative data from (**G**), including data from leukemia-free control mice. Data in **H** and **I** are pooled of 2 independent experiments with 2-4 mice/group and shown as mean ± SD.
